# Large‐scale GWAS in sorghum reveals common genetic control of grain size among cereals

**DOI:** 10.1111/pbi.13284

**Published:** 2019-11-11

**Authors:** Yongfu Tao, Xianrong Zhao, Xuemin Wang, Adrian Hathorn, Colleen Hunt, Alan W. Cruickshank, Erik J. van Oosterom, Ian D. Godwin, Emma S. Mace, David R. Jordan

**Affiliations:** ^1^ Queensland Alliance for Agriculture and Food Innovation (QAAFI) The University of Queensland Hermitage Research Facility Warwick Qld Australia; ^2^ Agri‐Science Queensland Department of Agriculture and Fisheries (DAF) Hermitage Research Facility Warwick Qld Australia; ^3^ Queensland Alliance for Agriculture and Food Innovation (QAAFI) The University of Queensland Brisbane Qld Australia

**Keywords:** grain size, GWAS, Sorghum, cereal crops, QTL, orthologues

## Abstract

Grain size is a key yield component of cereal crops and a major quality attribute. It is determined by a genotype’s genetic potential and its capacity to fill the grains. This study aims to dissect the genetic architecture of grain size in sorghum. An integrated genome‐wide association study (GWAS) was conducted using a diversity panel (*n* = 837) and a BC‐NAM population (*n* = 1421). To isolate genetic effects associated with genetic potential of grain size, rather than the genotype’s capacity to fill the grains, a treatment of removing half of the panicle was imposed during flowering. Extensive and highly heritable variation in grain size was observed in both populations in 5 field trials, and 81 grain size QTL were identified in subsequent GWAS. These QTL were enriched for orthologues of known grain size genes in rice and maize, and had significant overlap with SNPs associated with grain size in rice and maize, supporting common genetic control of this trait among cereals. Grain size genes with opposite effect on grain number were less likely to overlap with the grain size QTL from this study, indicating the treatment facilitated identification of genetic regions related to the genetic potential of grain size. These results enhance understanding of the genetic architecture of grain size in cereal, and pave the way for exploration of underlying molecular mechanisms and manipulation of this trait in breeding practices.

## Introduction

Cereal crops, including maize, rice, wheat, barley and sorghum, supply more than 75% of the calories consumed by humans (Sands *et al.*, 2009). They are critical to address global food security, which is under threat of population expansion and climate change. Among cereals, sorghum is an important source of food, fibre, feed and biofuel, and provides staple food for over 500 million people in the semi‐arid tropics of Africa and Asia. Cereal crops share a close ancestry, and strong gene co‐linearity among them supports comparative genomics approaches for exploring the genetic bases of agronomical traits at a cross‐species level (Bolot *et al.*, 2009). Grain size is a key yield component of grain yield in cereal crops and is a major quality attribute affecting planting, harvesting and processing activities (Tao *et al.*, 2017). A better understanding of the genetic basis of grain size, including underlying molecular mechanisms, will provide new targets for improving yield and grain quality in cereal breeding.

Grain size in cereals is a function of both the potential maximum grain size and the capacity of the plant to fill the grain. The weight of individual grains is determined by the rate and duration of grain filling. In sorghum, the grain filling rate is highly correlated with the ovary volume at anthesis, which in turn is associated with the size of the meristematic dome during early floret development (Yang *et al.*, 2009). While the potential maximum grain size is largely genetically predetermined, the capacity to fill the grain is strongly affected by assimilate availability (Gambín and Borrás, 2010). Grains are the key sink for carbon demand post‐anthesis, and the amount of available assimilate per grain is determined by grain number and assimilate supply, which are associated with a range of genetic and environmental factors (Gambín and Borrás, 2010; Wardlaw, 1990). Negative correlations between grain number and grain size are commonly observed in cereal crops (Acreche and Slafer, 2006; Peltonen‐Sainio *et al.*, 2007; Sadras, 2007; Tao *et al.*, 2018). In sorghum, grain number reduction at early stages of flowering has been observed to result in a larger grain weight (Sharma *et al.*, 2002). This indicates that demand for carbohydrate to fill grains exceeds the available supply.

In cereals, the genetic architecture of grain size is complex and involves numerous genes. In rice and maize, around 110 genes affecting grain size have been cloned (Tao *et al.*, 2017). Given the conservation of gene function among closely related cereal species, this presents opportunities for comparative genomics studies to investigate the genetics of grain size in other cereal crops using knowledge gained from gene cloning studies in maize and rice. The sorghum QTL atlas of Mace *et al. *(2018) details around 100 grain weight QTL in sorghum that have been identified through linkage analysis in 18 studies (Bai *et al.*, 2017; Boyles *et al.*, 2017; Brown *et al.*, 2006; Feltus *et al.*, 2006; Gelli *et al.*, 2016; Han *et al.*, 2015; Mocoeur *et al.*, 2015; Murray *et al.*, 2008; Paterson *et al.*, 1995; Phuong *et al.*, 2013; Rajkumar *et al.*, 2013; Rami *et al.*, 1998; Reddy *et al.*, 2013; Shehzad and Okuno, 2015; Spagnolli *et al.*, 2016; Srinivas *et al.*, 2009; Tao *et al.*, 2018; Tuinstra *et al.*, 1997). A further 12 markers associated with grain weight have been identified in three GWAS studies (Boyles *et al.*, 2016; Upadhyaya *et al.*, 2012; Zhang *et al.*, 2015). Given the number of genomic regions identified using standard genetic linkage mapping approaches, the limited number of marker–trait associations identified in the GWAS studies is likely due to the small population sizes employed in these studies (*n* = 242–390). All of the previous genetic studies on grain size in sorghum have focused on the dissection of the genetic basis of grain size under natural conditions, which also include environmental impacts and Genotype × Environment (G × E) interactions. However, minimization of variation in grain‐filling capacity could limit the environment effects on variation in grain size, and therefore facilitate identification of genetic factors underlying variation of this trait.

This study aims to (1) investigate the genetic architecture of grain size in sorghum by conducting an integrated GWAS in two large populations, a diversity panel of 837 individuals and a backcross‐nested association mapping (BC‐NAM) population of 1421 individuals, and (2) compare genetic loci affecting grain size identified in this study to those reported in rice and maize. To minimize variation in grain size caused by assimilate variability, a field treatment of removing half of the panicle during flowering time was imposed in this study in order to maximize assimilate availability for the remaining grains during grain filling.

## Results

### Minimizing environmental impact on grain size through the removal of half of the panicle during flowering time

In the DPGAT16 trial, a significant increase in average grain size of 8.54% was observed in half heads compared to full heads (Figure S2B). The magnitude of this increase varied significantly among genotypes, indicating the presence of genotypic variation in the degree of source limitation for grain filling in the full head (Figure S2C,D). Nonetheless, TKW of half‐head and full‐head treatments was highly correlated (*r* ^2^ ~ 0.70) (Figure S7). Since the greater average TKW of the half‐head treatment compared with the full‐head treatment indicated that this treatment was effective in removing some of the variation in grain size due to restrictions in grain‐filling capacity, data collected from plants with half‐head phenotypes were used in this study.

### Phenotypic variation of grain size

**Figure 1:**
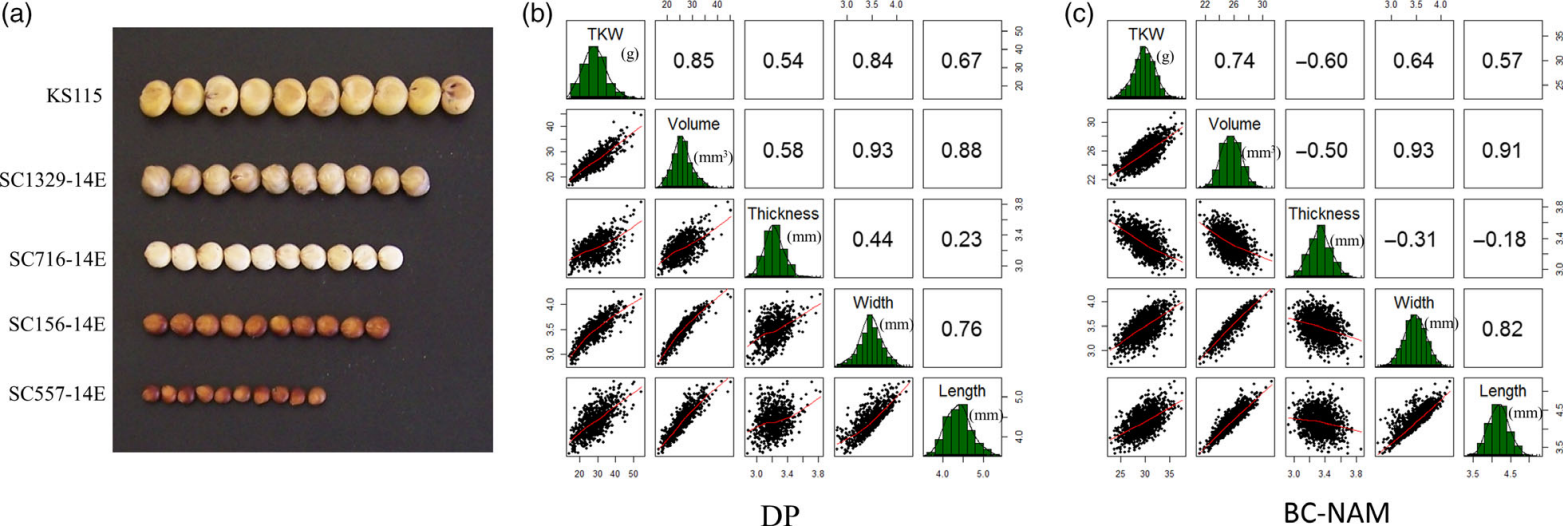
Phenotypic distribution and correlations of 5 grain size parameters in two populations. (a) Variation of grain size in sorghum. (b) Phenotypic distribution and correlations of 5 grain size parameters in the diversity panel. (c) Phenotypic distribution and correlations of 5 grain size parameters in BC‐NAM population. The plots on the diagonal show the phenotypic distribution of each trait. The values above the diagonal are pairwise correlation coefficients between traits, and the plots below the diagonal are scatter plots of compared traits.

Substantial variation in TKW and the volume, length, width and thickness of grains was observed in half‐head samples across the five trials and two populations (Tables 1 and 2, Figure 1). The diversity panel exhibited much broader variation in these grain size parameters than the BC‐NAM population, reflecting its larger genetic diversity. For example, in the trials conducted at Gatton in 2016, the range in TKW was 6.5–58.4 g in the diversity panel, compared to 16.9–44.5 g in the BC‐NAM population. All parameters had low levels of G × E interaction and were highly correlated across trials in both the diversity panel (*r* > 0.82, average *r* = 0.85) and the BC‐NAM population (*r* > 0.79, average *r* = 0.89) (Tables 1 and 2). Hence, the combined cross‐trial BLUPs were used for subsequent analyses of these parameters. The high cross‐trial correlations of the individual grain size parameters were consistent with their medium to high heritability (Tables 1 and 2).

**Table 1:** Summary of phenotypic data collected from half‐head samples across trials in the diversity panel

Trait	Trial	Trait mean	Range	H	Correlation between sites
TKW (g)	DPGAT16	28.8	6.5–58.4	0.79	0.87
TKW (g)	DPHER16	28.7	6.7–56.8	0.80	
Volume (mm^3^)	DPGAT16	26.2	8.4–55.0	0.67	0.85
Volume (mm^3^)	DPHER16	26.1	9.9–55.6	0.70	
Length (mm)	DPGAT16	4.41	2.93–5.85	0.68	0.88
Length (mm)	DPHER16	4.38	3.07−5.88	0.70	
Width (mm)	DPGAT16	3.50	2.29−4.74	0.65	0.84
Width (mm)	DPHER16	3.46	2.34−4.56	0.67	
Thickness (mm)	DPGAT16	3.22	2.4–4.05	0.67	0.82
Thickness (mm)	DPHER16	3.25	2.64–3.96	0.75	

H means broad‐sense heritability.

**Table 2:** Summary of the phenotypic data collected from half‐head samples across trials in the BC‐NAM population

Trait	Trial	Mean	Range	H	Correlations across trials		
					NAMGAT15	NAMGAT16	NAMHER15
TKW (g)	NAMGAT15	28.2	15.6–41.2	0.77	1	0.89	0.87
TKW (g)	NAMGAT16	29.9	16.9–44.5	0.53	0.89	1	0.98
TKW (g)	NAMHER15	30.8	13.9–45.6	0.66	0.87	0.98	1
Volume (mm^3^)	NAMGAT15	25.5	14.6–40.5	0.36	1	0.91	0.89
Volume (mm^3^)	NAMGAT16	25.3	15.0–38.7	0.37	0.91	1	0.81
Volume (mm^3^)	NAMHER15	25.7	15.4–36.4	0.41	0.89	0.81	1
Length (mm)	NAMGAT15	4.18	3.20–5.29	0.41	1	0.92	0.91
Length (mm)	NAMGAT16	4.17	3.29–5.31	0.45	0.92	1	0.85
Length (mm)	NAMHER15	4.18	3.31–5.07	0.40	0.91	0.85	1
Width (mm)	NAMGAT15	3.47	2.58–4.32	0.29	1	0.91	0.87
Width (mm)	NAMGAT16	3.47	2.65–4.26	0.39	0.91	1	0.79
Width (mm)	NAMHER15	3.48	2.76–4.15	0.29	0.87	0.79	1
Thickness (mm)	NAMGAT15	3.32	2.71–3.72	0.54	1	0.93	0.89
Thickness (mm)	NAMGAT16	3.31	2.85–3.69	0.41	0.93	1	0.86
Thickness (mm)	NAMHER15	3.35	2.9–3.78	0.59	0.89	0.86	1

H means broad‐sense heritability.

All five grain size parameters displayed near‐normal distributions in both populations (Figure 1b,c), which, in combination with the medium to high heritability and low G × E observed, indicated that variation in the parameters was controlled by multiple genetic loci. High correlations among the five grain size parameters were also observed in both populations, implying a high degree of shared genetic control among them. The principal components analysis determined that 3 PCs accounted for the majority (>85%) of the variation in the grain size parameters observed across multiple trials in both populations (Figures S3 and S4). The direction of the correlations between traits was positive in all contrasts with the exception of grain thickness, which displayed negative correlations with the other four grain size parameters in the BC‐NAM population (*r* = −0.39), as opposed to positive correlations (*r* = +0.44) in the diversity panel.

**Figure 2:**
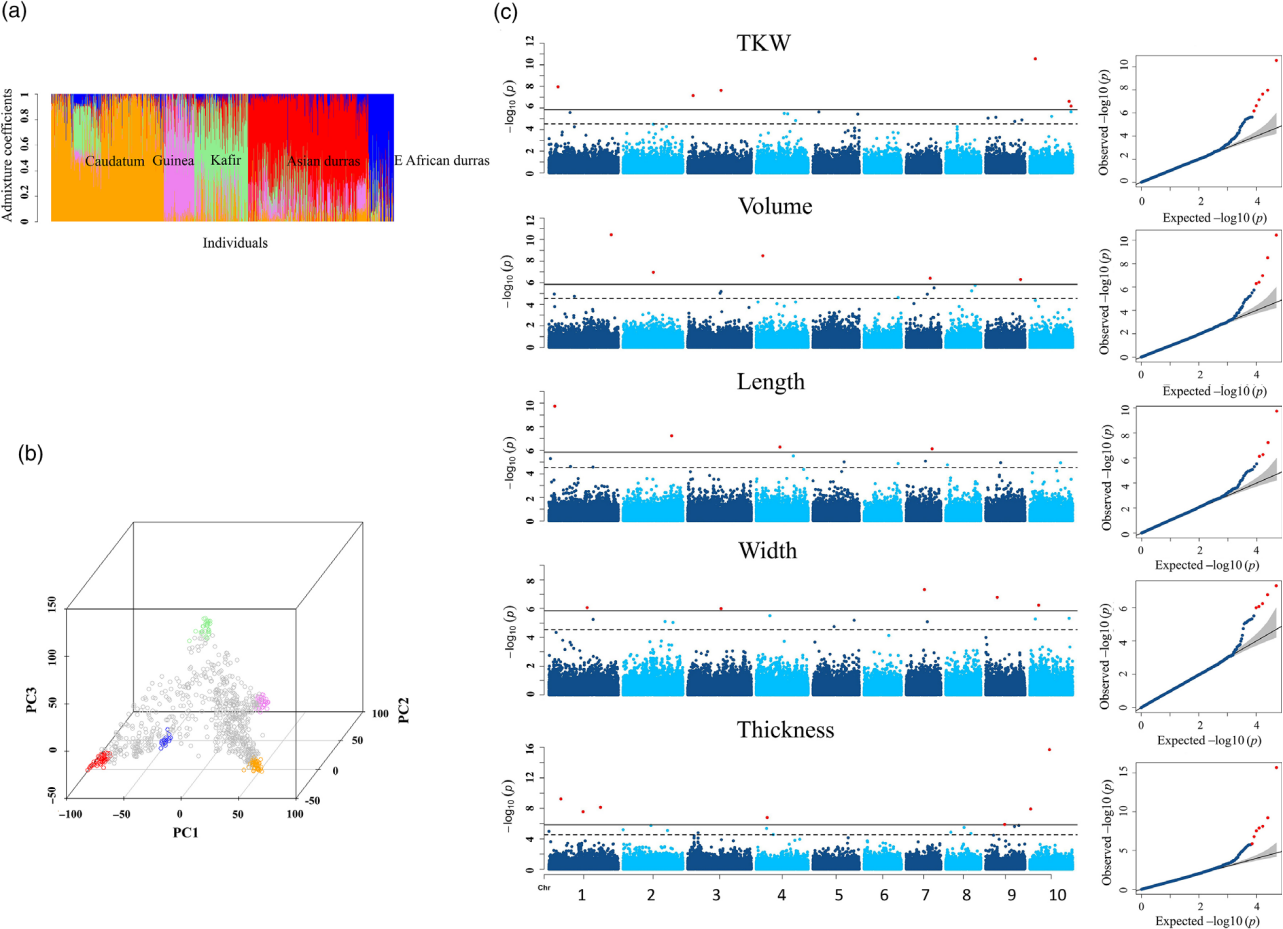
GWAS of 5 grain size parameters in the diversity panel. (a) Population structure of the diversity panel. Orange represents caudatum, violet represents guinea, light green represents kafir, red represents Asian durras, and blue represents East African durras. (b) PCA of the diversity panel, dots in colour indicate representatives for each racial group. (c) GWAS results were displayed in Manhattan plots and Q‐Q plots. Significant threshold (solid black line) and suggest threshold (dash black line) were 1.45E‐06 and 2.91E‐05, respectively, according to GEC test.

To investigate possible correlations between racial groups and grain size parameters in sorghum, structure analysis of the diversity panel was conducted, which revealed 5 groups that corresponded to different racial groups of sorghum (Figure 2a, Table S4). The classification of 5 racial groups within the diversity panel was further validated in a PCA(Figure 2b). All grain size parameters showed significant differences among these racial groups (ANOVA, *P*‐value < 0.05), with representatives of the caudatum and guinea racial groups having the largest and heaviest grains and East African durras the smallest and lightest (Table S7).

### Identification of genetic loci associated with variation of grain size

**Figure 3:**
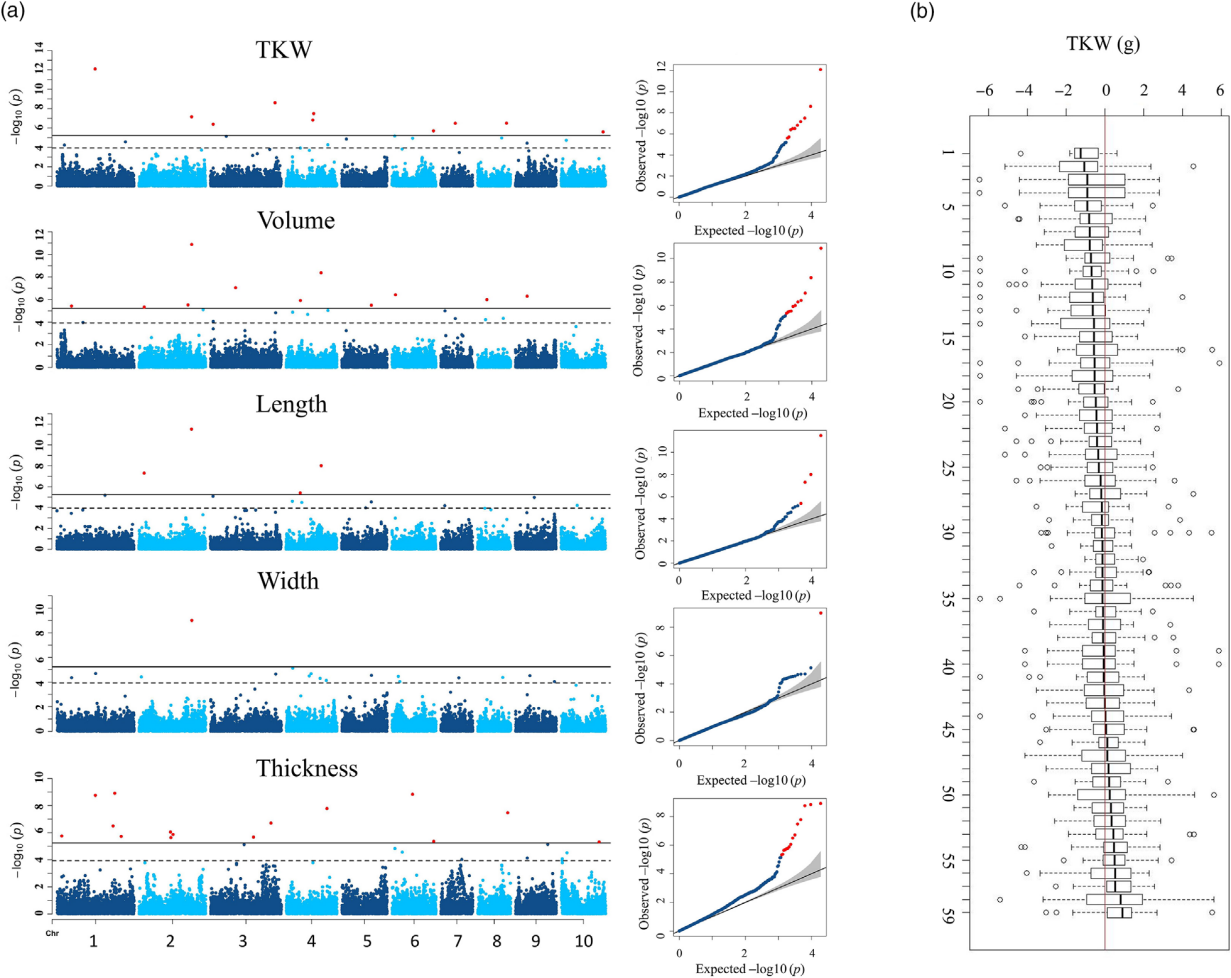
GWAS of 5 grain size parameters in the BC‐NAM population. A) GWAS results were displayed in Manhattan plots and Q‐Q plots. Significant threshold (solid black line) and suggest threshold (dash black line) were 5.83E‐06 and 1.17E‐04, respectively, according to GEC test. B) Effects of exotic alleles of grain size QTL relative to elite recurrent parents in the BC‐NAM population. Each box plot illustrates the effects of exotic alleles of a grain size QTL across BC‐NAM families. Within each family, effects of a QTL were calculated as difference between average of the exotic allele and average of allele of the recurrent parental line. Fifty‐nine QTL identified in BC‐NAM were ordered based on the median effect of the exotic alleles. Correspondence between numbers on x‐axis and QTL identity is provided in Table S16.

In the half‐head panicles from the diversity panel, GWAS identified 82 marker–trait associations, with 69 SNPs significantly associated with between 1 and 4 of the grain size parameters (Figure 2c, Figure S8 and Table S8). It is worth noting that the FarmCPU approach produces Manhattan plots consisting of individual, or small numbers of markers, at QTL locations in comparison with the more traditional GWAS output consisting of multiple significant markers at QTL resembling the Manhattan skyline (Liu *et al*, 2016). On average, LD in the diversity panel decayed to half of its maximum value at ~ 20 kb, and to the background level at ~ 200 kb, equivalent to 1 cM (Figure S9a). Thus, a 1‐cM window was used to cluster these 69 SNPs into 51 QTL. The majority (>80%) of these 51 QTL were significantly associated with multiple grain size parameters (Table S9). However, phenotypic variation explained by individual QTL was generally small and the majority of QTL explained < 5% of phenotypic variation each. In the BC‐NAM population, 119 significant marker–trait associations were identified with 94 SNPs associated with between 1 and 4 grain size parameters (Figure 3a, Figure S10 and Table S10). Because the extent of LD was greater in the BC‐NAM than in the diversity panel (Figure S9b), a 2‐cM window was used to cluster these associated SNPs into 59 QTL regions. Similar to observations for the diversity panel, the vast majority (>80%) of the QTL were significantly associated with multiple grain size parameters and phenotypic variation explained by individual QTL was generally small, with the majority of QTL explaining < 5% of the phenotypic variation of a given grain size parameter (Table S11). Within the BC‐NAM, it was possible to observe the distribution of allelic effects of the exotic parents compared to the recurrent parent (Figure 3b). In general, alleles were observed that were both larger and smaller than the elite parent, which was supportive of the presence of multiple alleles at the majority of QTL.

A comparison of the QTL identified initially in both populations separately revealed 29 QTL in common, with 22 QTL being unique to the diversity panel and 30 QTL unique to the BC‐NAM population (Figure S11), resulting in 81 QTL identified in total across populations. These 81 QTL were distributed across all 10 sorghum chromosomes (Table 3). Further examination revealed that the majority of the population‐specific QTL (20 out of 22 QTL unique to the diversity panel and 24 out of 30 QTL specific to BC‐NAM population) were significantly associated with grain size parameters in the alternative population using the candidate region‐based association analysis (Table S12).

**Table 3:** Summary of the grain size QTL identified in the diversity and BC‐NAM populations

QTL ID	Chr	P‐start	P‐end	G‐start	G‐end	Traits affected		Overlap
						DP	BC‐NAM	
qGS1.1	1	1197421	1197421	3.18	3.18	_VL__	TVLW_	Y
qGS1.2	1	3900109	5918319	10.63	13.66	TVLWH	TVLWH	Y
qGS1.3	1	6907688	7312727	16.89	18.27	TVLWH	TVLWH	Y
qGS1.4	1	9747369	9819285	25.45	25.45	TVLWH	_____	Y
qGS1.5	1	12725797	13016063	31.69	32.84	TV__H	TVLWH	Y
qGS1.6	1	17427840	17638047	43.49	43.60	TVLW_	T___H	Y
qGS1.7	1	21451374	21347457	46.73	46.68	TVLW_	____H	N
qGS1.8	1	53202019	53936221	59.15	60.07	TV__H	TVLWH	N
qGS1.9	1	58396003	58396003	67.93	67.93	TVLWH	T____	Y
qGS1.10	1	62888113	62968722	94.75	94.83	TVLW_	TVLWH	N
qGS1.11	1	67567101	67567101	116.76	116.76	_VLWH	_V__H	Y
qGS1.12	1	68155796	68372498	123.06	125.38	____H	TVLWH	Y
qGS1.13	1	72940563	72940563	138.72	138.72	TVL_H	TV__H	N
qGS1.14	1	76174795	76174795	159.22	159.22	T____	T_L_H	Y
qGS2.1	2	127186	1044744	0.00	0.10	____H	TVLW_	N
qGS2.2	2	2902033	2977717	13.86	13.93	TV_WH	TVLWH	N
qGS2.3	2	56922554	56922554	88.63	88.63	TV_WH	_VLW_	N
qGS2.4	2	57497352	57512278	93.16	93.17	TV_WH	____H	N
qGS2.5	2	58950144	58950144	101.44	101.44	TVLWH	____H	N
qGS2.6	2	59194748	59194748	106.68	106.68	_VL__	TVLWH	N
qGS2.7	2	62351000	62351000	134.53	134.53	T____	__LWH	N
qGS2.8	2	66339425	66573634	148.73	149.23	___W_	____H	Y
qGS2.9	2	67764171	68007224	152.70	152.89	TV_WH	TVLW_	Y
qGS2.10	2	68868784	69048935	158.62	159.86	TVLW_	TVLW_	N
qGS2.11	2	70379906	70379906	166.28	166.28	TVLW_	TVLW_	Y
qGS2.12	2	73494298	74155670	181.05	182.44	TVL_H	TVLWH	Y
qGS3.1	3	1678233	1678922	4.04	4.05	_____	TVLW_	N
qGS3.2	3	3597439	3597439	11.06	11.06	TVLWH	TVLWH	Y
qGS3.3	3	13099674	13099674	48.31	48.31	_VLWH	TVLWH	Y
qGS3.4	3	27806521	50560393	56.79	57.86	TV_WH	T_LWH	Y
qGS3.5	3	54907966	54907966	70.31	70.31	TV_WH	_VLW_	Y
qGS3.6	3	58595865	58595865	102.06	102.06	T__WH	T_L_H	Y
qGS3.7	3	70185379	70185379	149.33	149.33	TV_WH	TV_WH	Y
qGS3.8	3	71510515	71802973	154.35	155.80	TVLWH	TVLWH	Y
qGS4.1	4	4223866	4229836	27.71	27.75	TV_WH	_VLW_	N
qGS4.2	4	4559807	4559807	29.99	29.99	TVLWH	_____	N
qGS4.3	4	7553732	7553732	55.81	55.81	____H	___W_	Y
qGS4.4	4	13834052	49497026	71.37	75.54	TVLW_	TVLWH	Y
qGS4.5	4	51365107	52404329	82.14	84.01	TVLWH	TVLWH	Y
qGS4.6	4	54000525	54337830	94.49	94.71	TVLW_	TVLWH	Y
qGS4.7	4	57705089	59916793	106.83	107.52	_VL__	TVLW_	Y
qGS4.8	4	61396436	61772402	121.78	123.63	TVLWH	TVLWH	Y
qGS4.9	4	62264856	62264856	126.15	126.15	TV_WH	TVLWH	Y
qGS5.1	5	3301237	3575185	22.87	23.25	TVLWH	TVLW_	Y
qGS5.2	5	62745836	63339533	73.38	73.89	TVLWH	TVLW_	Y
qGS6.1	6	2325346	2491448	28.20	30.03	_____	TVLWH	N
qGS6.2	6	3407665	3407665	32.68	32.68	_____	TVLWH	Y
qGS6.3	6	39213141	43198219	50.50	52.75	_____	TVLWH	Y
qGS6.4	6	49521704	49663694	89.11	89.27	T_L__	____H	Y
qGS6.5	6	55753427	55753427	127.38	127.38	_VL__	T_L__	N
qGS6.6	6	59304932	59994056	160.95	162.94	TVLW_	TVLWH	Y
qGS7.1	7	2508289	2508289	27.97	27.97	TVLWH	TV_W_	N
qGS7.2	7	52373156	43625689	72.22	72.45	TV_WH	TV_W_	Y
qGS7.3	7	52822173	54307590	74.35	75.24	__LW_	TV_W_	Y
qGS7.4	7	55675271	55675271	78.67	78.67	TVLWH	___W_	N
qGS7.5	7	58298370	58728728	89.41	91.13	_VLWH	____H	N
qGS7.6	7	60263992	60263992	106.63	106.63	TVLW_	___W_	Y
qGS8.1	8	2625914	2683449	30.27	30.34	T____	T_L_H	Y
qGS8.2	8	5558686	5997613	58.76	59.32	TVLW_	TVLWH	Y
qGS8.3	8	10712721	10712721	67.33	67.33	TV_WH	_V___	Y
qGS8.4	8	58069324	58373421	87.75	88.00	TVLWH	_VLW_	N
qGS8.5	8	59615859	59873063	97.46	99.35	TV__H	TV_WH	N
qGS8.6	8	60306822	60394245	102.58	103.12	TVLW_	TVLWH	N
qGS8.7	8	61382934	61382934	109.44	109.44	T___H	_V___	N
qGS8.8	8	61773908	61868146	111.98	112.60	_____	TVLWH	N
qGS8.9	8	61977230	61977230	118.53	118.53	_____	TVLWH	N
qGS9.1	9	6653224	6653224	62.22	62.22	TVLW_	T_L_H	N
qGS9.2	9	7768372	15253577	67.33	69.39	__L__	TVLWH	Y
qGS9.3	9	42995650	44651836	75.64	76.01	____H	TVLWH	Y
qGS9.4	9	47977415	47977415	84.70	84.70	T___H	__L__	N
qGS9.5	9	52757898	52757898	104.82	104.82	T___H	T___H	N
qGS9.6	9	53947707	53959840	108.19	108.23	T__W_	TVLWH	Y
qGS9.7	9	55387311	55578639	112.20	112.74	T___H	TVLWH	N
qGS9.8	9	56628436	56628436	115.46	115.46	TVLWH	TV_WH	N
qGS9.9	9	58508803	58508803	130.88	130.88	TV_WH	TVLW_	N
qGS10.1	10	677400	777135	11.60	13.22	_V__H	____H	N
qGS10.2	10	2875254	5478495	30.84	34.32	TVLWH	T____	Y
qGS10.3	10	12131889	35248936	56.00	58.44	TVLWH	__L_H	Y
qGS10.4	10	51630406	51630406	67.65	67.65	TVLWH	TVLW_	Y
qGS10.5	10	56447045	56993830	89.00	90.54	____H	T___H	N
qGS10.6	10	58653863	60172927	102.30	103.56	TVLWH	TVLW_	N

Chr indicates the chromosome on which the QTL is located. P‐start provides the physical coordinate, based on v3.1.1 of the sorghum genome assembly, of the start of the QTL CI. P‐end provides the physical coordinate of the end of the QTL CI. G‐start provides the predicted cM genetic linkage position of the start of the QTL CI based on the sorghum consensus map (Mace *et al*, 2009). G‐end provides the predicted cM genetic linkage position of the end of the QTL CI. The traits affected columns indicate the traits significantly associated with the QTL in diversity panel (DP) and BC‐NAM population, respectively. The five traits were recorded as TVLWH, where T represents TKW, V represents volume, L represents length, W represents width, and H represents thickness. The inclusion of the letters for a particular QTL means that the corresponding traits are affected by the QTL in the corresponding population. Missing letters in the order of TVLWH (represented as ‘_’) mean that the corresponding traits are not affected by the QTL in corresponding population. The Overlap column indicates whether the QTL overlaps with previously published grain size QTL in sorghum. N means the QTL is not overlapped with previously published grain size QTL, while Y means the QTL is overlapped with previously published QTL.

The genetic basis of grain size has been the focus of 18 previous studies in sorghum, which used 21 bi‐parental populations and reported 85 grain weight QTL (Table S5). Nearly three quarters (60) of these previously reported QTL co‐located with QTL identified in this study. A significant overlap between the QTL identified in this study and grain size SNPs identified in rice (Huang *et al.*, 2012) was observed, with 60% of the rice grain size SNPs identified within a 1‐cM window of the sorghum QTL identified in this study (*P*‐value < 0.05, chi‐square test), and with 80% of the rice grain size SNPs identified within 5 cM (Table S13). A similar trend was found when comparing with kernel size SNPs identified in maize (Liu *et al.*, 2019), with 26% and 70% of maize kernel size SNPs identified within 1‐cM and 5‐cM window of sorghum QTL identified in this study (Table S13).

### Candidate genes for grain size QTL

To identify putative causative genes underlying the grain size QTL, 109 genes affecting grain size that were reported in rice and maize were collated and 111 orthologues in sorghum were identified (Table S6; Table S14). The candidate genes were enriched in the grain size QTL; 13 of the 111 candidate genes were co‐located with grain size QTL (*P*‐value < 0.05, chi‐square test), and 32 were identified within a 1‐cM window (*P‐*value < 0.05, chi‐square test) (Table S15). Some of the negative correlations between grain number and grain size are likely due to variation in the genotypes’ capacity to fill the grain as observed in this study. Given the treatment imposed in the current study to minimize variation due to grain filling capacity, we would expect that the previously reported candidate genes that have been shown to exert opposite effects on grain size and grain number would be less represented within our set of QTL. To explore this hypothesis, the candidate genes were divided into 3 groups based on whether they had been reported to exert opposite effects on grain number and grain size in the species in which they were cloned (i.e. whether the gene was associated with bigger grains and lower grain number). This approach identified 21 genes that showed opposite effects on grain size and grain number, 37 genes that did not show opposite effects and 53 genes where this information was not reported (Table S15). It was found that the candidate genes that were reported to have an opposite effect on grain number and grain size were underrepresented in the overlap with the QTL from this study (Table S15).

One of the candidate genes, *SbGS3,* the orthologue of the rice grain size gene *GS3* (Mao *et al.*, 2010), was co‐located with *qGS1.9,* a QTL identified in both the diversity and BC‐NAM populations, for grain length, weight, volume, width and thickness. In the diversity panel, one SNP was identified that caused a C to A change in the fifth exon of *SbGS3*, turning a cysteine codon (TG**C**) to a premature stop codon (TG**A**), resulting in the 198 amino acid protein being truncated at the 140th amino acid. However, the most significant SNP identified for *qGS1.9* was 11.48kb (0.01cM) away from *SbGS3*, rather than the large‐effect SNP causing truncation at the 140th amino acid. This may have been due to the low MAF (22 out of 837 individuals carried the premature stop codon allele) and the similar genetic background for the individuals carrying the premature stop codon allele. Transcriptomics expression data (Makita *et al.*, 2015) showed that *GS3* had comparable expression patterns in sorghum and rice, with high expression levels observed in the panicle and minimal expression in other tissue types covering a range of temporal and spatial variation (Figure S12a). Additional transgenic experiments, using RNAi, have demonstrated that inhibiting the expression of *SbGS3* increased grain weight by 9.4% on average in sorghum (Trusov et al., pers. comm.). Both positive and negative allelic effects from non‐recurrent parental lines were observed in the BC‐NAM compared with the recurrent parental line, R931945‐2‐2, indicating the existence of multiple alleles of *SbGS3* in the population (Figure S12b).

## Discussion

Grain size is one of the most critical traits of cereal crops due to its direct contribution as a yield component, its importance as a quality attribute and its contribution to fitness stemming from its impact on reproductive rates, emergence and establishment (Tao *et al.*, 2017; Westoby *et al.,* 1992). This study represents one of the largest and most comprehensive investigations of the genetic basis underlying this trait in any cereal via a joint GWAS analysis of grain size in sorghum using a diversity panel (*n* = 837) and a BC‐NAM population (*n* = 1421). The population size is the largest of all GWAS studies on grain size in any cereal crop to our knowledge. As a result, a total number of 81 grain size QTL were identified. Enrichment of grain size candidate genes within these QTL was observed, and significant overlap between these 81 QTL and SNPs associated with grain size in rice and maize was found, highlighting common genetic control of this trait among cereals. In addition, the treatment of removing half the panicle during flowering facilitated minimization of variation of grain size due to assimilate variability, and identification of genetic regions related to the genetic potential of grain size rather than the capacity to fill the grain. Therefore, these results provide an enhanced understanding of the genetic basis underlying grain size in sorghum and pave the way for exploration of its underlying molecular and physiological mechanisms in cereal crops and its manipulation in breeding practices.

### The relationship between grain size and grain number in sorghum is mainly driven by genes acting before grain filling

A negative correlation between grain size and number is widely observed in cereals. The nature of this correlation is complex (Sadras, 2007) and is partially driven by the fact that potential maximum grain size and grain number are established before grain filling and by the availability of assimilates to enable grains to reach their genetic potential (Gambín and Borrás, 2010; Yang *et al.*, 2009). In this study, the half‐head treatment resulted in an average increase in TKW of 8.54%, with significant variation among genotypes. The average change in grain weight is in line with previous estimates of sorghum’s capacity to compensate for loss of grain (Sharma *et al.*, 2002). Grain weight in the full‐head treatment explained ~70% of the genetic variation in grain size in the half‐head treatment, indicating that while assimilate availability is a significant factor influencing grain size, most of the genetic factors driving the correlation between grain size and number in sorghum are acting prior to grain filling. Future yield gains may require that this association is better understood to determine whether the negative association can be broken at some or all QTL.

### Shared genetic control of grain size dimensions

Sorghum grains vary in terms of length, thickness, width, volume and weight. Substantial genetic variation was observed for all of these parameters in both populations. Cross‐environment correlations of these grain size parameters within the same population were very high, and the heritability of the traits was also moderate to high, indicating strong genetic control as previously observed (Prado *et al.*, 2014; Tao *et al.*, 2018). These findings, combined with a normal distribution of the phenotypes, indicate that these parameters are likely to be controlled by many genes with small to moderate effects. In addition, strong correlations were observed between the different grain size parameters in the same population, indicating they have a high degree of common genetic control. This was expected for parameters such as volume and weight and their correlations with the various measured dimensions, since the traits are linked mathematically. In contrast to positive correlations between other grain size parameters in both populations, the grain thickness trait was negatively correlated with the other 4 parameters in the BC‐NAM population but positively correlated with these parameters in the diversity panel. This is likely due to the different genetic backgrounds of the two populations. The diversity panel has a good representation of sorghum’s racial groups (Thurber *et al.*, 2013). Although the BC‐NAM also provides a good representation of the genetic variation of the racial groups of sorghum, because of the population structure (backcross derived based on a single reference parent), the genetic composition of each line is strongly biased towards the genetic background of the elite recurrent parent, which is primarily of *caudatum* origin.

### High‐confidence genetic architecture of grain size revealed through independent multi‐population GWAS

Population size is a key factor affecting the power of a GWAS study, as is the need to control for type I and type II errors (Visscher *et al.*, 2017). The two populations used in this study, taken individually, are among the largest published to date in any cereal crop on grain size. This size, coupled with the capacity to independently verify putative QTL in the alternative population, has provided us with a substantial advantage over previously published studies. The diversity panel contains representatives of most of the racial groups in the sorghum gene pool and is characterized by its fast LD decay. In contrast, the BC‐NAM population consists of BC_1_‐derived lines sharing ~80% of their genomes with the elite reference parent (Jordan *et al.*, 2011), which provides an opportunity to assess exotic alleles in the genetic background of an elite inbred. The BC‐NAM population has lower genetic diversity, a more balanced structure and greater LD and therefore potentially more power to detect QTL. The joint use of these complementary populations has also provided a more comprehensive understanding of the genetic architecture of grain size, and ready‐to‐use information on the effect of QTL alleles in elite breeding material.

The high number (81) of QTL identified in this study and their relatively small individual effects highlight the genetic complexity of grain size. Over half of the QTL identified in this study (46) were co‐located with QTL identified in previous sorghum studies (Table 3) with < 30% of the previous QTL not detected in this study (Table S5). Care must be taken with interpreting this result however, since the small population sizes of the previous studies limited their power to detect QTL and resulted in large confidence intervals to the extent that they often encompassed a number of the QTL detected in this study. In addition, two of the previous studies identified QTL from crosses between wild and domestic sorghum (Paterson *et al.*, 1995; Tao *et al.*, 2018), which may not have been detected in our study as wild species were not included in either population, and none of the other studies attempted to minimize variation in the capacity to fill grain. Hence, it is likely that some of the QTL previously detected may not be segregating in our study.

Our study was internally consistent, with 90% of the QTL supported by evidence from the two independent populations either via co‐located QTL or with support from the candidate region analysis, resulting in only 8 population‐specific QTL being identified. Given that the two populations were independent and differed in genetic composition, power and LD, the high correspondence between the two studies suggests that we identified the majority of the loci for grain size in cultivated sorghum.

### Significant allelic diversity exists in grain size QTL

The BC‐NAM population provided us an opportunity to investigate variation in the effects of different QTL alleles in an elite genetic background. Our results suggest the presence of multiple alleles at the majority of the QTL with effects of the non‐recurrent parental alleles that were both greater and smaller than the recurrent parent allele observed at most loci (Figure 3b). The distribution of the allele effects provides interesting insights into selection for the grain size trait in an elite breeding programme. There was weak tendency for the elite parental alleles to contribute to larger grain size compared to the exotic parent. This is similar to an earlier study of flowering time in the same population where positive and negative allele effects relative to the recurrent parental allele were similarly distributed (Mace *et al.*, 2013). The lack of bias observed in the current study suggests modern sorghum breeding with its focus on increasing grain yield has not selected for grain size. The lack of strong selection for grain size by modern breeders is interesting and suggests a potential physiological restriction to increasing grain yield by selection for larger grain. This is consistent with the observation that much of the progress in enhancing grain yield in modern breeding of cereals has been achieved by selection for increased grain number rather than grain size. Clearly, further research is required before breeders attempt to increase grain yield by introducing alleles for larger grain size. However, given the range of allele effects, it is likely some opportunities exist to simultaneously improve grain yield and grain size, for example through manipulation of the duration of grain filling (Yang *et al.*, 2010).

### Correspondence of loci controlling grain size among cereals

Sorghum shares close ancestry with maize and rice, with orthologous genes from these species commonly sharing the same function (Bolot *et al.*, 2009; Paterson *et al.*, 1995; Zhang *et al.*, 2015). A significant association was found between the locations of the QTL in this study and GWAS signals for grain size in rice (Huang *et al.*, 2012) and maize (Liu *et al.*, 2019), and orthologous of cloned grain size genes from rice and maize. Both findings strongly support a common genetic architecture underlying this trait across these cereals. This provides opportunities for breeders to exploit information from other cereals, as genes identified from large‐scale GWAS in one species can be targeted for allele mining or diversity creation via gene editing in another species.

As an example, the gene *SbGS3*, the orthologue of the cloned gene *Grain Size 3* in rice, was found to control variation of grain size in sorghum. *SbGS3* showed a similar expression pattern as *GS3* in rice with high expression in early inflorescence, indicating the same role in grain development. Further evidence from transgenic experiments has shown that inhibiting the expression of *SbGS3* increased grain weight by 9.4% on average in sorghum (Trusov et al., pers. comm.). It has also been reported that the orthologue of *GS3* in maize, *ZmGS3*, was involved in kernel development (Li *et al.*, 2010). GS3 contains four domains: an OSR domain in the N terminus, a transmembrane domain, a TNFR/NGFR family cysteine‐rich domain and a VWFC in the C terminus (Mao *et al.*, 2010). In rice, overexpression of a truncated cDNA sequence of *GS3* where the VWFC domain is deleted produced shorter grains (Mao *et al.*, 2010). The premature stop change of *SbGS3* caused a truncation of the VWFC domain. Further investigation is required to see whether comparable effects caused by the truncation occur in sorghum.

## Materials and methods

### Plant material

Two populations, together comprising over 2000 individuals, were used in this study: a diversity panel (DP, *n* = 837) (Table S1) and a BC‐NAM consisting of 30 interrelated families (*n* = 1421) (Figure S1a; Table S2). The diversity panel has around 225 genotypes in common with the US sorghum association panel. It consists predominantly of lines developed by the Sorghum Conversion Program conducted by Texas Agricultural Experiment Station, which took diverse sorghum lines from the world collection and converted tall, late or photoperiod‐sensitive sorghums from the tropics into short, early or photoperiod‐insensitive types that could be used by breeders in temperate regions (Rosenow *et al.*, 1997). The programme involved repeated backcrossing to the exotic line combined with selection for height and maturity in temperate environments. The resulting material has been reported to contain >4% genome introgression from the temperate donor with the remainder from the exotic parent and has a much narrower range of height and maturity than the original exotic lines (Thurber *et al.*, 2013).

The BC‐NAM population was previously developed by the Department of Agriculture and Fisheries (DAF), Queensland, Australia, and described in Jordan *et al. *(2011). In summary, each BC‐NAM family was produced by crossing a single elite parent (R931945‐2‐2 or R986087‐2‐4‐1) with a diverse, exotic line (the non‐recurrent parent) and backcrossing the resulting F_1_ to the elite parent to produce a large BC_1_F_1_ population. Variable numbers of plants were selected from each BC_1_F_1_ family, and using single‐seed descent, >5 generations of self‐pollination were generated to produce BC_1_F_6_ and beyond. During generation advance, selection was imposed for height and maturity of the recurrent parental type.

The 30 BC‐NAM families used in the current study are listed in Table S2. In total, 27 unique non‐recurrent parental lines and 2 elite recurrent parental lines were used for population development. These included 10 diverse lines from advanced breeding programmes and 17 landraces, 7 of which were converted to temperate adaptation through the Sorghum Conversion Program (Stephens *et al.*, 1967). Fourteen of the 27 non‐recurrent parental lines were also included in the diversity panel (Figure S1b).

### Field Trials and phenotypic evaluation

A total of 5 field trials were planted at Hermitage Research Facility (HER), Warwick, Queensland, Australia (28°12′S, 152°5′E, 470 m above sea level), and Gatton Research Facility (GAT), Gatton, Queensland, Australia (27°33′S, 152°20′E, 94 m above sea level), during the Australian summer cropping seasons of 2014/15 and 2015/16. Two trials of the diversity panel were grown in the 2015/16 season at Hermitage (DPHER16) and Gatton (DPGAT16). The BC‐NAM were planted in three trials at Hermitage in 2014/15 (NAMHER15) and Gatton in 2014/15 (NAMGAT15) and 2015/16 (NAMGAT16). All trials were planted between November and February, using a row‐column design with partial replication. Each plot consisted of two 6‐m rows with a row spacing of 0.75 m. Differences in plant height within a plot were minimal. Different numbers of genotypes were grown in each trial due to seed availability (Table S3). The soils at all sites were all highly fertile vertisols, 300 kg/ha of urea was applied (prior to planting at Warwick and via fertigation at Gatton) to ensure nitrogen supply throughout plant growth. All experiments were monitored for pest insects. No insecticidal control was necessary, but viral insecticide was applied prior to flowering at Warwick to ensure no damage by lepidopteran larvae. Standard agronomic practices and pest‐control practices were applied.

A treatment of removing half of each of two panicles in each plot was imposed when each panicle commenced flowering, before significant grain development had occurred (Figure S2a). Once physiological maturity was reached, the remaining half panicle (hereafter, referred as half head) was hand‐harvested and threshed using a mechanical threshing machine. In DPGAT16, a full‐head panicle in each plot was also harvested and threshed for comparison with the half‐head panicles from the same plot. An aspirator was used to remove any debris from each sample before measurements were taken. Although sorghum grain is typically tending towards spherical, considerable phenotypic variation in length, width and thickness does exist. Therefore, grain size parameters, including thousand kernel weight (TKW), and the length, width, thickness and volume of grains were measured using SeedCount SC5000 (Next Instruments, Condell Park, NSW, Australia) and a digital balance.

### Statistical analysis

Due to the large number of genotypes included in this study, partial replication was used in all trial designs (Cullis *et al.*, 2006), with 30 % of the genotypes replicated two or more times and the remaining 70 % represented by single plots. The total number of plots in each trial ranged from 880 to 1521, with the total number of genotypes planted in each trial ranging from 658 to 1164 (Table S3). A customized design was used to minimize spatial error effects within each trial. The concurrence of genotypes and populations across the two seasons allowed the DP and BC‐NAM trials to be analysed as two multi‐environment trials (METs) for each of the five measured grain size parameters, comprising of two trials for the DP and three for the BC‐NAM. Each MET was analysed by fitting a linear mixed model using the package ASReml (Butler *et al.*, 2009) and the R statistical software. Each model consisted of a fixed effect for the targeted trait at each trial, random effects for genotype within trial and spatial error for each individual trial (Smith *et al.*, 2001). The G × E interaction was analysed by fitting a third‐order factor analytic structure to the Trial × Genotype interaction; in this case, the structure also modelled the 3‐way interaction between site, trait and genotype. The analysis resulted in genetic variances for each trial and loading values representing factor analytic loadings.

Generalized repeatability estimates were calculated for grain size parameters using the method proposed by Cullis *et al. *(2006). High correlations were observed among grain size parameters, and hence, a principal components analysis was conducted to further investigate the interrelationships among TKW and grain length, width and thickness (Figures S3 and S4). Because it is possible that a constructed trait such as a principal component may explain the data more effectively, the main principal components were used as additional traits in the association analysis.

### Genotyping and imputation

The 2 populations were genotyped using medium‐ to high‐density genome‐wide SNPs provided by Diversity Arrays Technology Pty Ltd (www.diversityarrays.com). DNA was extracted from bulked young leaves of five plants from a plot of each genotype in the 2 populations using a previously described CTAB method (Doyle, 1987). The DNA samples were then genotyped using DArTseq, which involves complexity reduction of the genomic DNA to remove repetitive sequences using methylation‐sensitive restriction enzymes (HpaII, MseI) prior to sequencing of the most informative representations of genomic DNA on next‐generation sequencing platforms (Illumina, HiSeq 2500) (Edet *et al.*, 2018). The sequence data generated were then aligned to version v3.1.1 of the sorghum reference genome sequence (McCormick *et al.*, 2018) to identify SNPs (single nucleotide polymorphisms).

In total, 111,089 SNPs were identified in the diversity panel and 31,478 SNPs in the BC‐NAM population. The overall proportion of missing data reported in the raw genotypic data sets was approximately 10% and 5% for the diversity panel and BC‐NAM, respectively. Individual SNP markers with >50% missing data were removed from further analysis, and the remaining missing values were phased and imputed using Beagle v4.1 (Browning and Browning, 2016). An average imputation accuracy of 96% was achieved across both populations.

### Population statistics, GWAS analysis and QTL identification

Pairwise linkage disequilibrium (LD) (*r* ^2^) was calculated using PopLDdecay (Zhang *et al.*, 2018) for the diversity and the BC‐NAM. The population structure in the diversity panel was analysed used LEA in R (François, 2016). The structure analysis identified five groups that corresponded to 4 racial groups (kafir, caudatum, guinea, durras of Asian origin and durras of East African origin) based on previous racial information and geographical origin for a subset of lines. Each individual line with ≥ 95 % of genetic identity from one of the five identified groups was designated as a representative of the corresponding racial group (Table S4). The remaining lines were considered as admixtures of multiple groups.

For the GWAS analysis, imputed SNP data sets for both populations were filtered for minor allele frequency (MAF) >0.01. The final number of SNPs used for GWAS was 49,586 for the diversity panel (Figure S5) and 18,932 for the BC‐NAM population (Figure S6). For the diversity panel, a principal components analysis (PCA) was conducted using a pruned SNP data set to control for population structure. Marker pruning was conducted using PLINK (Purcell *et al.*, 2007) with a sliding window of 100 SNPs, a step size of 50 SNPs and *r^2^* of 0.5. For the BC‐NAM population, pedigree was used to control population structure. GWAS was performed using FarmCPU (Liu *et al.*, 2016) for both populations for the five measured grain size parameters and derived PCs. Thresholds for defining significant and suggestive associations were set using the Bonferroni correction based on the number of independent tests, calculated using the GEC software package (Li *et al.*, 2012). Marker–trait associations were then identified using a two‐step process. Firstly, SNPs were identified that were significantly associated with grain size parameters and derived PCs in both populations separately (*P* < 1.45E‐06 for the diversity panel; *P* < 5.83E‐06 for the BC‐NAM). Secondly, SNPs were identified that were only suggestively associated with grain size parameters and derived PCs in one population (*P* < 2.91E‐05 for the diversity panel, 1.17E‐04 for the BC‐NAM), but that had either suggestive or significant support in the second population and were also within a specified distance of the original SNP (<1 cM in the diversity panel and < 2 cM in the BC‐NAM population, calculated from the sorghum genetic linkage consensus map; Mace *et al.*, 2009). Identified marker–trait associations were further clustered into QTL regions based on the predicted genetic positions of SNPs from the sorghum consensus map across traits within each population (Mace *et al.*, 2018). Finally QTL in common between the two populations were combined based on a 2‐cM window. The effects of identified QTL on all grain size parameters in each population were assessed using a linear model, with population structure accounted for by PCs for the diversity panel and by pedigrees in the BC‐NAM population.

A candidate region‐based approach was used to further investigate associations between population‐specific QTL and grain size parameters in the alternative population. SNPs within a 0.5‐cM window of a population‐specific QTL were extracted from the alternative population. To examine associations between SNPs in the candidate regions and the grain size parameters, GLM in TASSEL 5.0 was run, using either PCA in the diversity panel or pedigree in the BC‐NAM population as a covariate to control for population structure (Bradbury *et al.*, 2007). The Bonferroni correction (0.05/number of markers) was used to control for false positives.

Using the BC‐NAM population, allele effects of the exotic parent were estimated compared to the recurrent parent. At each QTL, the numbers of exotic alleles with larger and smaller values than the recurrent parent were determined, and a test was conducted to assess whether the numbers of positive and negative alleles relative to the recurrent parent varied. Families with < 20 individuals were excluded from this analysis.

### Collection of previously reported grain weight QTL

Eighty‐five grain weight QTL were collated from previously published studies (Table S5). The locations of these QTL were projected onto both the sorghum reference genome v3.1.1 and the sorghum consensus map following methods described by Mace *et al. *(2009, 2018). To be conservative, QTL with confidence interval> 20 cM were excluded during this step.

### Candidate gene analysis

Candidate genes for grain size in sorghum were identified using the methods described by Tao *et al. (*2017). Firstly, genes functionally determined to control grain size in rice and maize were collated through a literature search (Table S6). Subsequently, corresponding sorghum orthologous of grain size genes in rice and maize were identified using a combination of syntenic and bidirectional best hit (BBH) approaches. Syntenic gene sets among rice, maize and sorghum were downloaded from PGDD (http://chibba.agtec.uga.edu/duplication/) to identify syntenic orthologues of known grain size genes from rice and maize. Local blast was performed to identify best BLAST hits of pairs of genes from two genomes, using BLASTP.

## Author contributions

D.J., E.M. and I.G. conceived and designed the experiments; Y.T., X.Z., X.W. and A.C. collected data; Y.T., C.H., A.H., E.O., D.J. and E.M. analysed data; Y.T. wrote the manuscript; E.M., E.O., I.G. and D.J. revised the manuscript. All authors read and approved the final manuscript.

## Funding information

This work was supported by the Australian Research Council (ARC) Discovery Project DP14010250.

## Conflict of Interest

The authors declare that the research was conducted in the absence of any commercial or financial relationships that could be construed as a potential conflict of interest.

## Supporting information


**Figure S1** PCA of BC‐NAM.
**Figure S2** Field treatment and its effect on grain size.
**Figure S3** Bi‐plots show PCA analysis of grain size parameters measured in the diversity panel.
**Figure S4** Bi‐plots show PCA analysis of grain size parameters traits measured in BC‐NAM.
**Figure S5** Distribution of SNPs across sorghum genome in the diversity panel.
**Figure S6** Distribution of SNPs across sorghum genome in BC‐NAM.
**Figure S7** Correlation of grain size between HH and FH.
**Figure S8** Manhattan plots and Q‐Q plots show GWAS analysis of PCs derived from grain size parameters in the diversity panel.
**Figure S9** LD decay of the diversity panel (a) and the BC‐NAM (b).
**Figure S10** Manhattan plots and Q‐Q plots show GWAS analysis of PCs derived from grain size parameters in BC‐NAM.
**Figure S11** The overlap of grain size QTL identified in the diversity panel and the BC‐NAM population.
**Figure S12** *SbGS3 *(Sobic.001G341700) controls grain size in sorghum.

**Table S1** List of the diversity panel.

**Table S2** Summary of the BC‐NAM.

**Table S3** Summary of field experiments.

**Table S4** Racial group breakdown of the diversity panel.

**Table S5** List of reported grain size QTL in previous studies.

**Table S6** List of reported grain size genes in rice and maize.

**Table S7** Effect of population structure on grain size. Statistical significance was assessed using one‐way analyses of variance (ANOVAs) followed by Tukey’s HSD tests for multiple comparisons

**Table S8** Marker Trait associations in the diversity panel. Physical positions of SNPs were according to *Sorghum bicolor* v3.1.1.

**Table S9** Effects of QTL in the diversity panel

**Table S10** Marker Trait associations in BC‐NAM. Physical positions of SNPs were according to *Sorghum bicolor* v3.1.1.

**Table S11** Effects of QTL in BC‐NAM.

**Table S12** Tests of associations between population‐specific QTL with grain size parameters in the alternative population.

**Table S13** Comparison of grain size regions identified in rice and maize with grain size QTL identified in this study.

**Table S14** List of grain size candidate genes in sorghum.

**Table S15** Summary of candidate genes within close vicinities of grain size QTL.

**Table S16** Correspondence between numbers on x‐axis of Figure 3B and identities of QTL identified in BC‐NAM.
