# Supplementary material for: Large‐scale GWAS in sorghum reveals common genetic control of grain size among cereals
Source: Plant Biotechnol J. 2019 Nov 11;18(4):1093–105. doi: 10.1111/pbi.13284 (PMC7061873; doi:10.1111/pbi.13284)
Supplement: Supplementary file 1 — Figure S1 PCA of BC‐NAM. Figure S2 Field treatment and its effect on grain size. Figure S3 Bi‐plots show PCA analysis of grain size parameters measured in the diversity panel. Figure S4 Bi‐plots show PCA analysis of grain size parameters traits measured in BC‐NAM. Figure S5 Distribution of SNPs across sorghum genome in the diversity panel. Figure S6 Distribution of SNPs across sorghum genome in BC‐NAM. Figure S7 Correlation of grain size between HH and FH. Figure S8 Manhattan plots and Q‐Q plots show GWAS analysis of PCs derived from grain size parameters in the diversity panel. Figure S9 LD decay of the diversity panel (a) and the BC‐NAM (b). Figure S10 Manhattan plots and Q‐Q plots show GWAS analysis of PCs derived from grain size parameters in BC‐NAM. Figure S11 The overlap of grain size QTL identified in the diversity panel and the BC‐NAM population. Figure S12 SbGS3 (Sobic.001G341700) controls grain size in sorghum. [file PBI-18-1093-s016.pptx]

## Slide 1
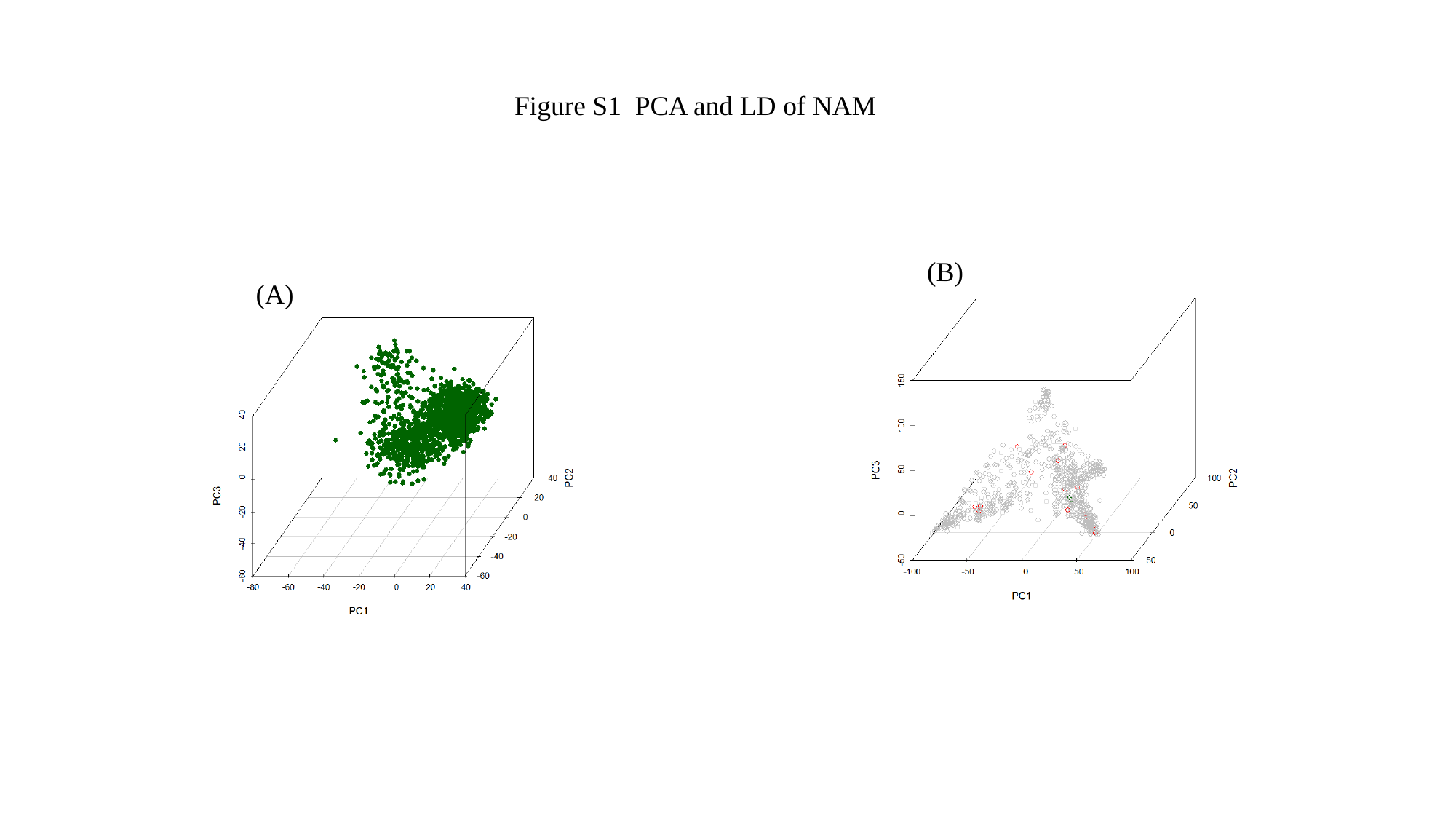

Figure S1 PCA and LD of NAM
(B)
(A)

## Slide 2
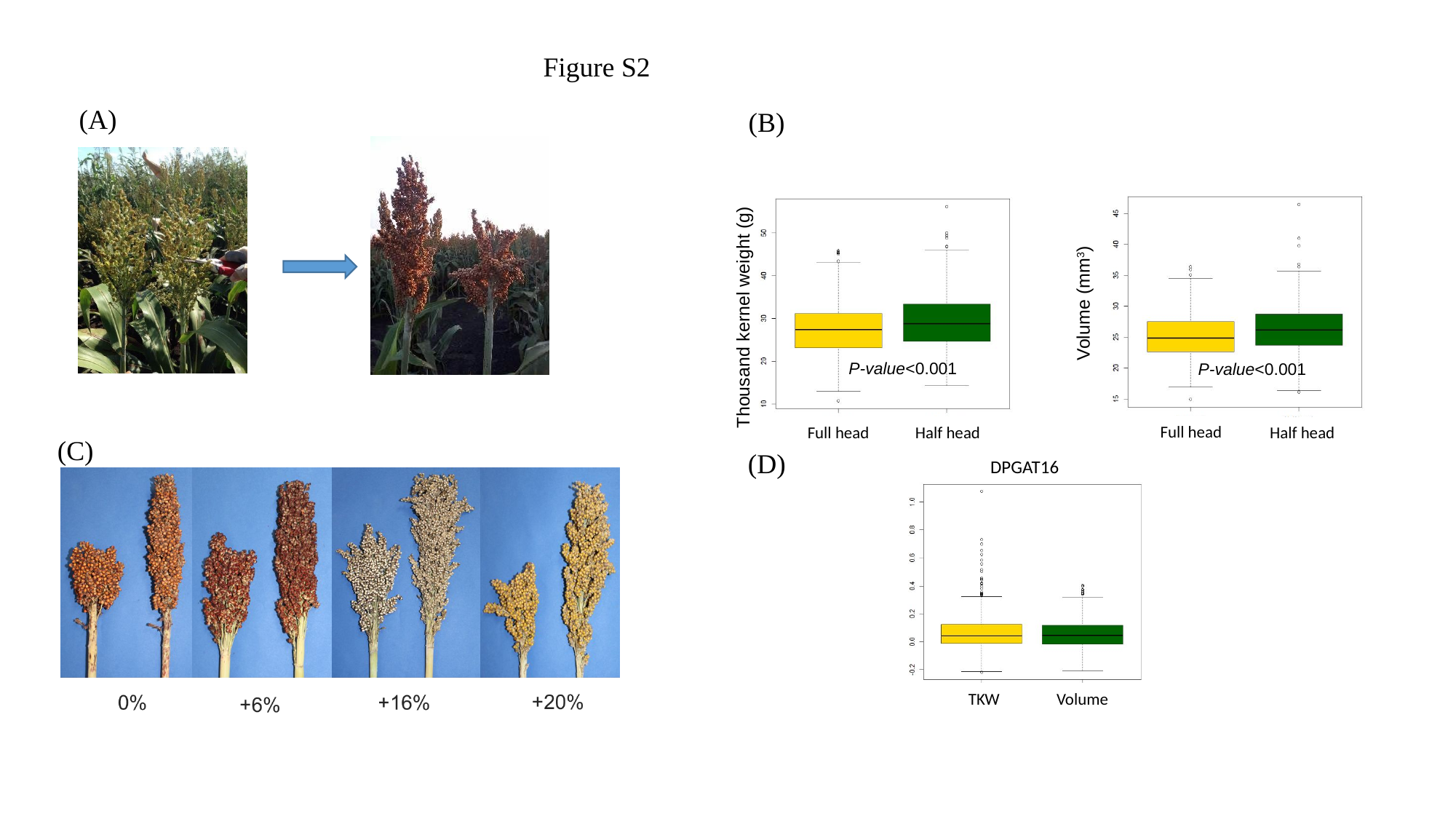

Figure S2
(A)
(B)
Volume (mm3)
Thousand kernel weight (g)
P-value<0.001
P-value<0.001
Full head
Full head
Half head
Half head
(C)
(D)
DPGAT16
TKW
Volume

## Slide 3
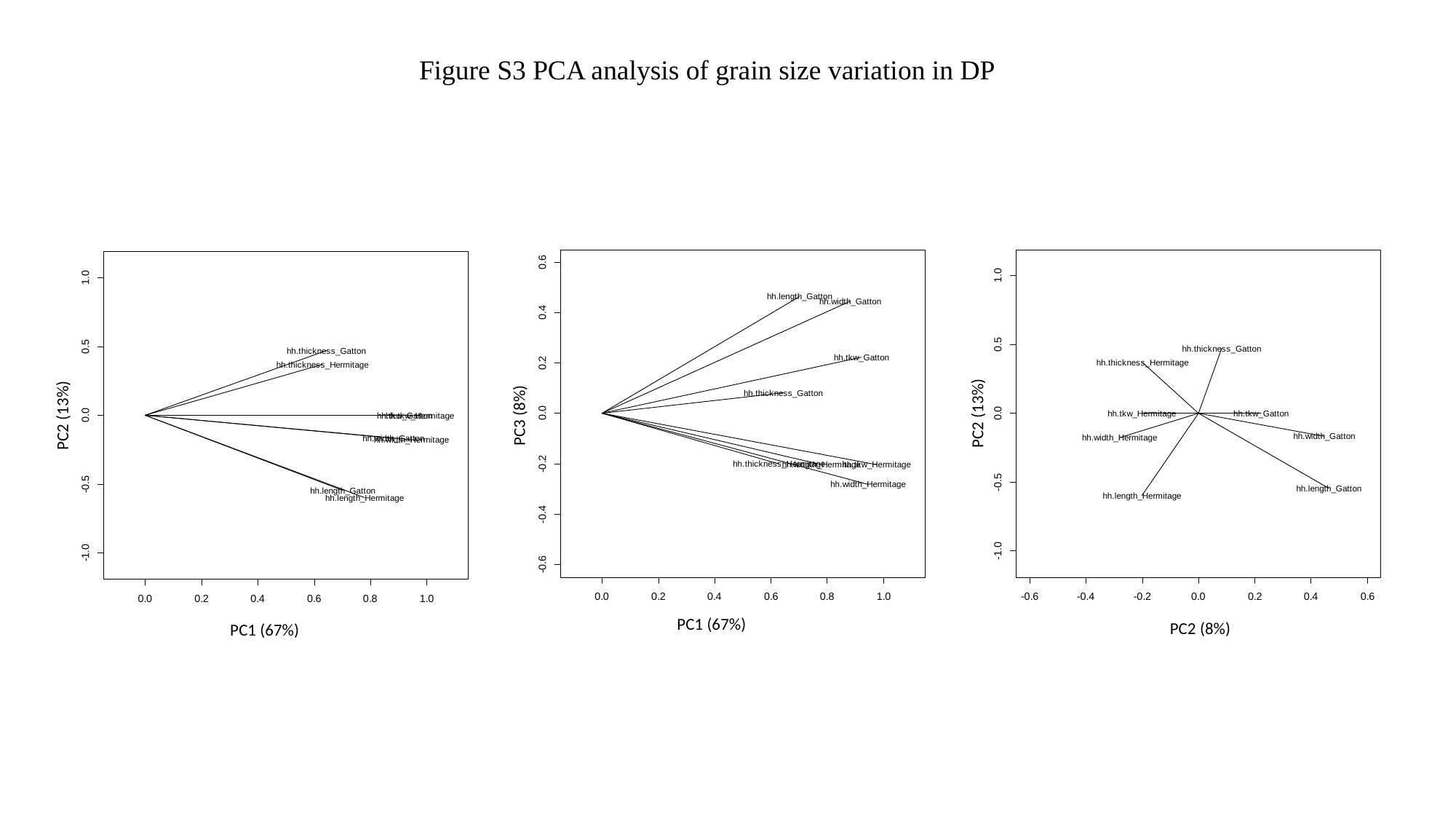

Figure S3 PCA analysis of grain size variation in DP
PC3 (8%)
PC1 (67%)
PC2 (13%)
PC2 (8%)
PC2 (13%)
PC1 (67%)

## Slide 4
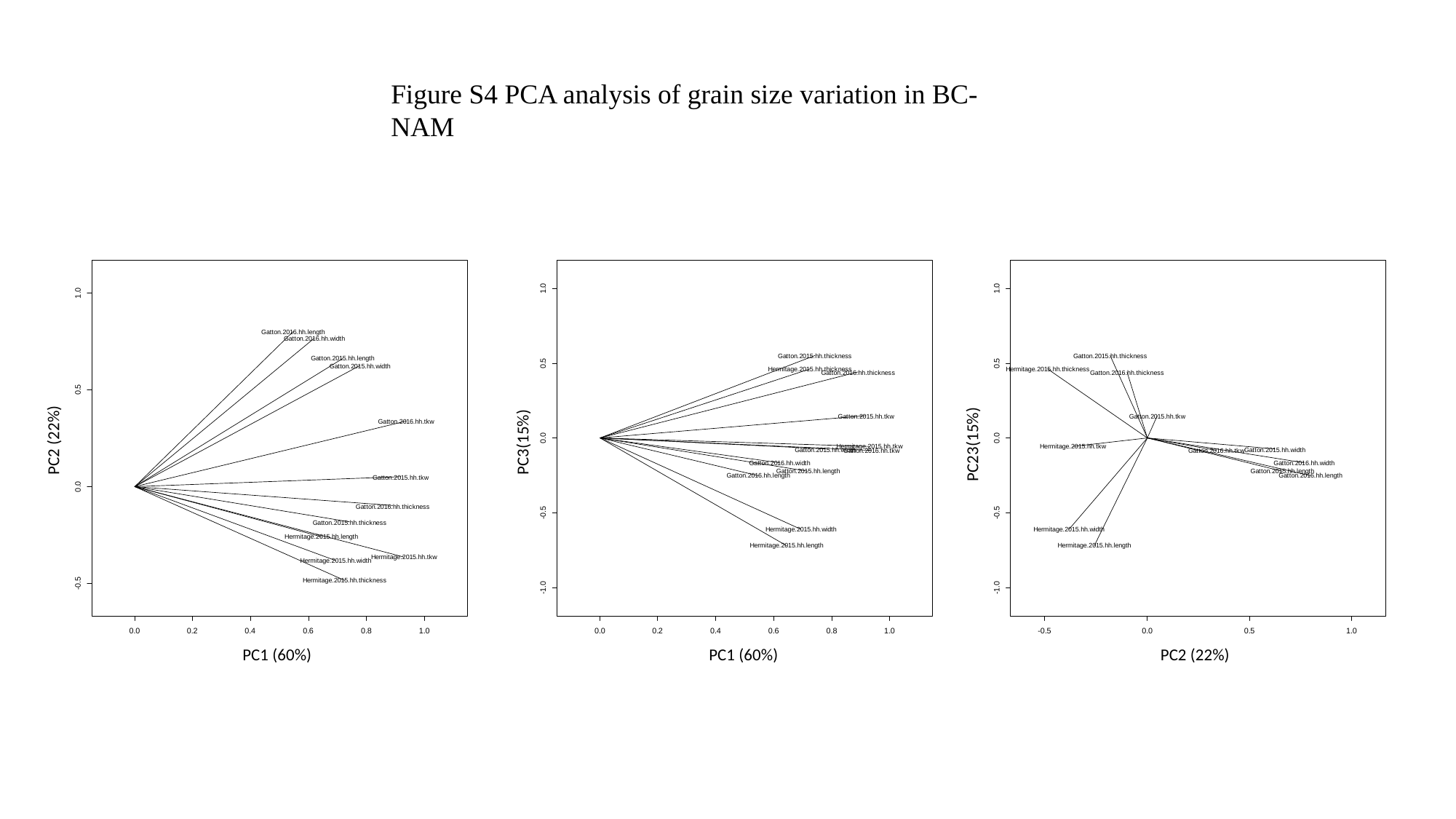

Figure S4 PCA analysis of grain size variation in BC-NAM
PC3(15%)
PC1 (60%)
PC23(15%)
PC2 (22%)
PC2 (22%)
PC1 (60%)

## Slide 5
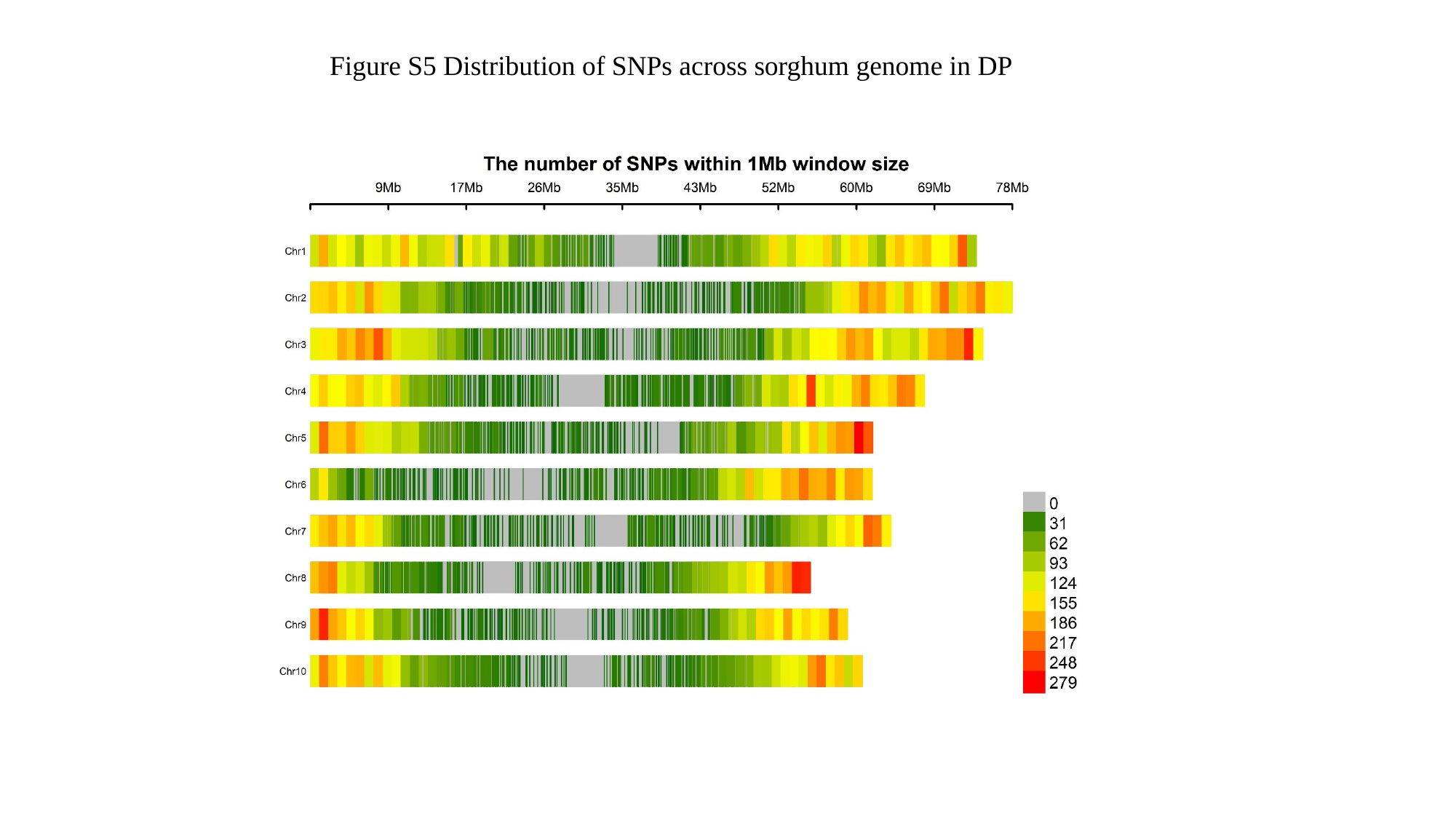

Figure S5 Distribution of SNPs across sorghum genome in DP

## Slide 6
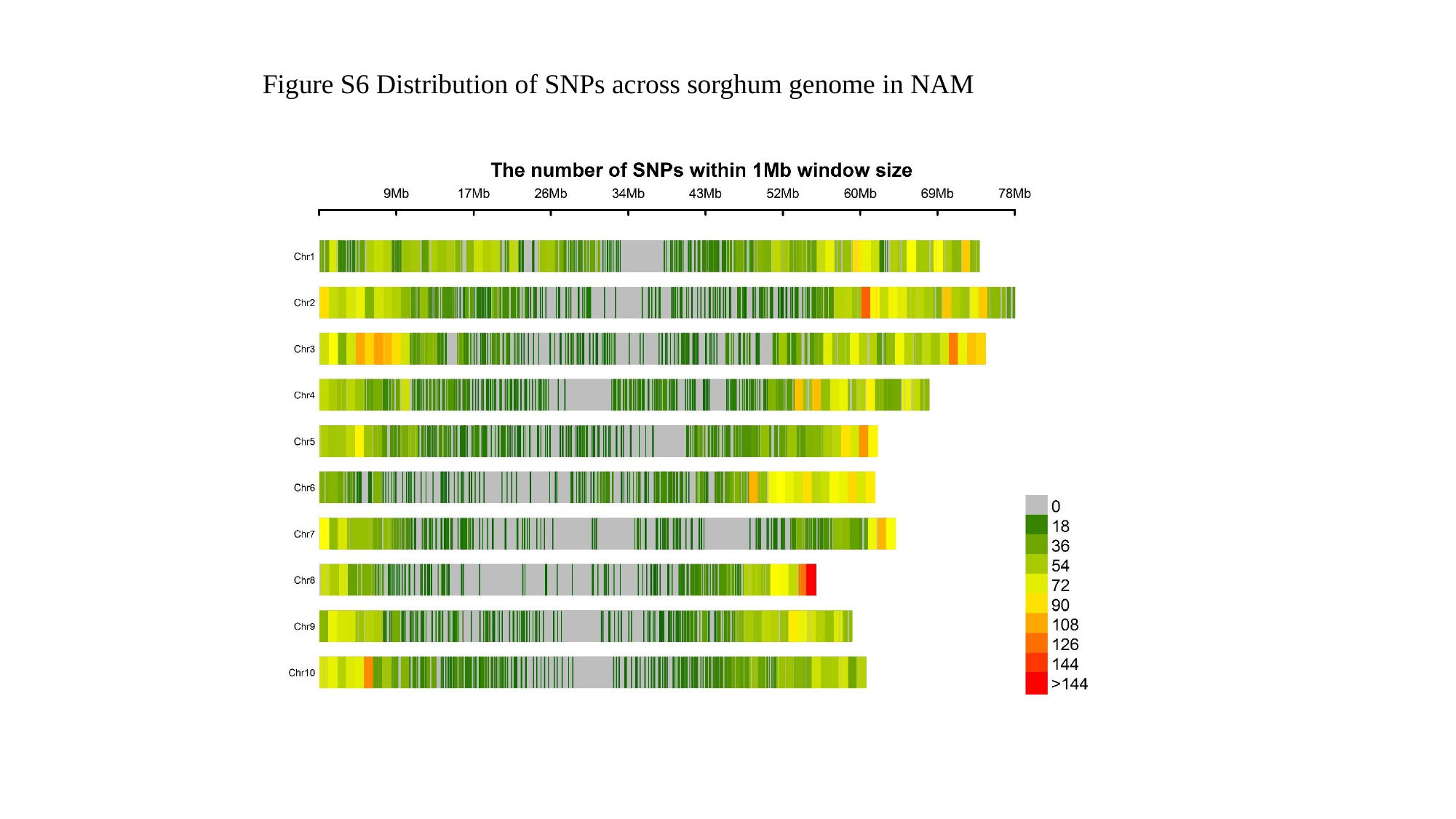

Figure S6 Distribution of SNPs across sorghum genome in NAM

## Slide 7
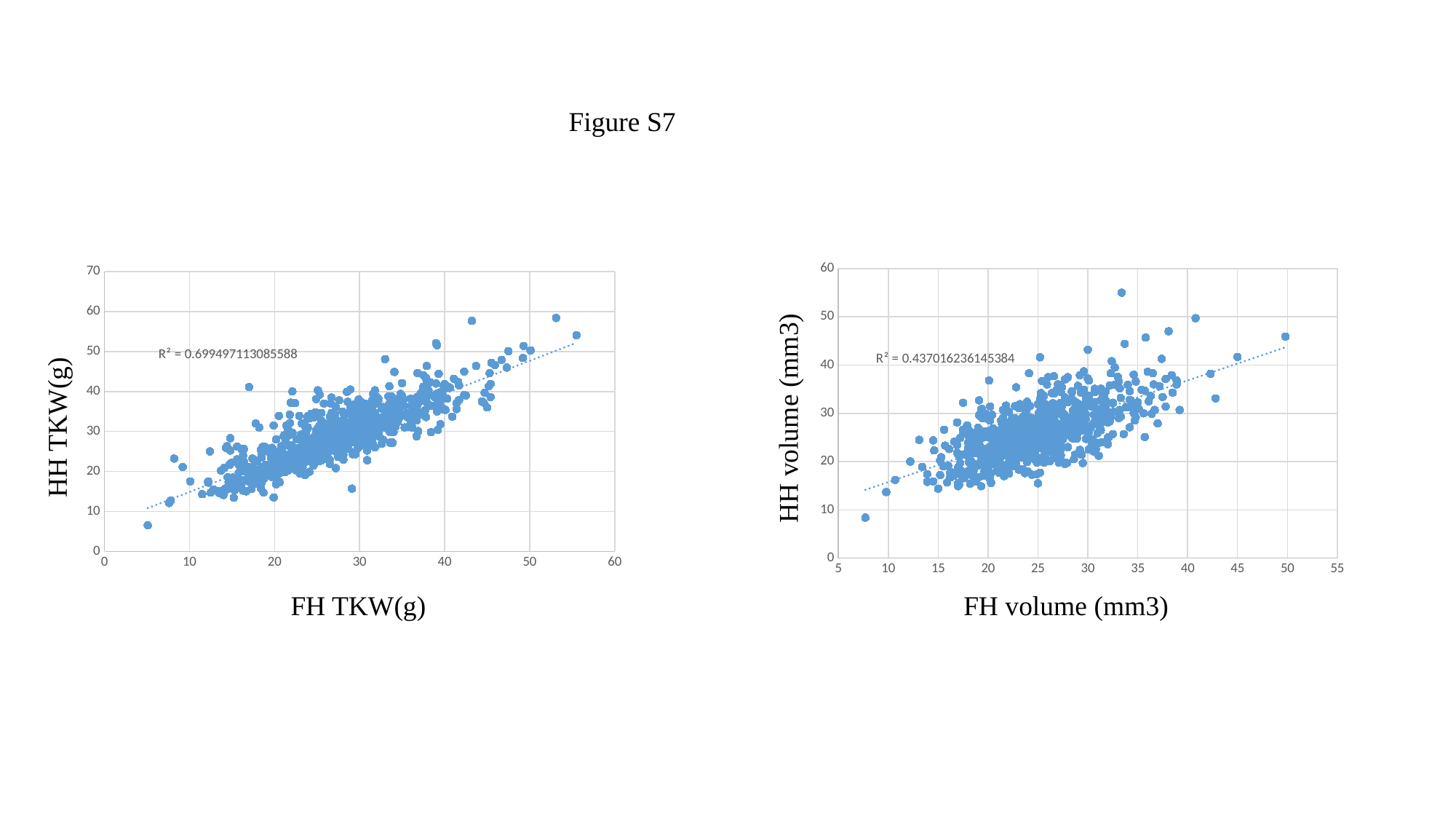

Figure S7
### Chart
| Category | HH-TKW |
|---|---|
### Chart
| Category | HH-Volume |
|---|---|HH volume (mm3)
HH TKW(g)
FH TKW(g)
FH volume (mm3)

## Slide 8
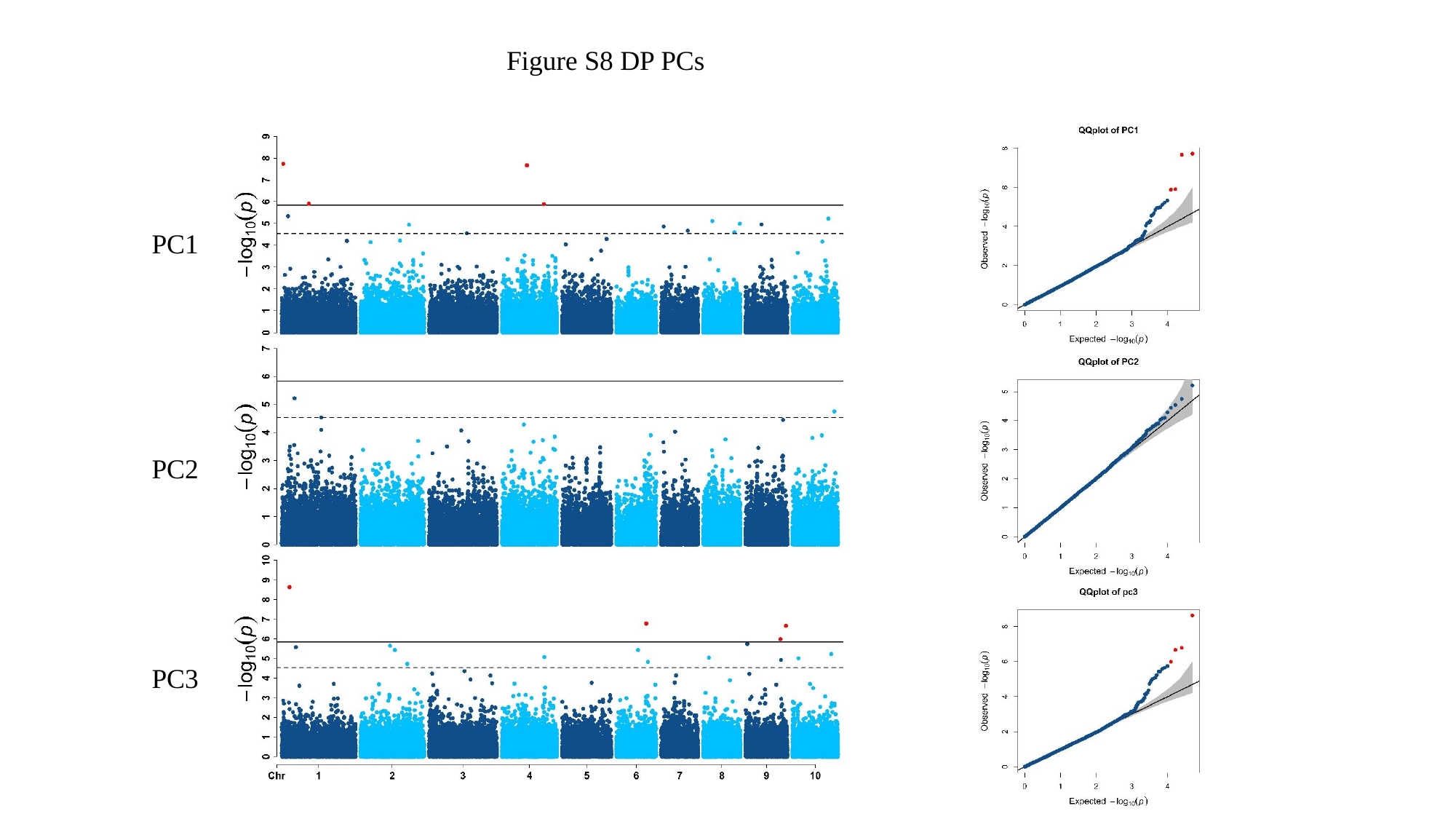

Figure S8 DP PCs
PC1
PC2
PC3

## Slide 9
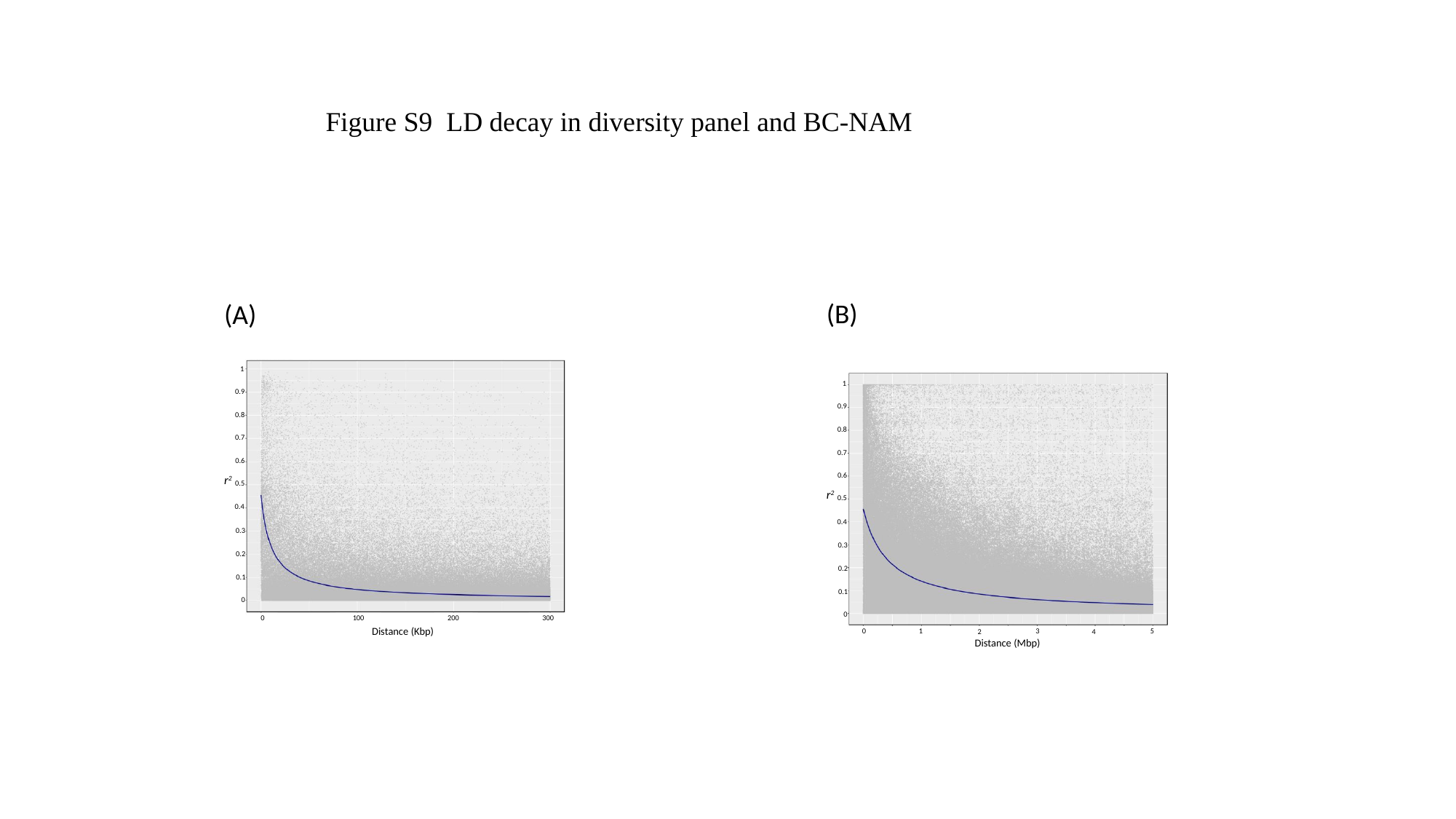

Figure S9 LD decay in diversity panel and BC-NAM
(B)
(A)
1
0.9
0.8
0.7
0.6
r2
0.5
0.4
0.3
0.2
0.1
0
300
0
100
200
Distance (Kbp)
1
0.9
0.8
0.7
0.6
r2
0.5
0.4
0.3
0.2
0.1
0
5
0
3
1
2
4
Distance (Mbp)

## Slide 10
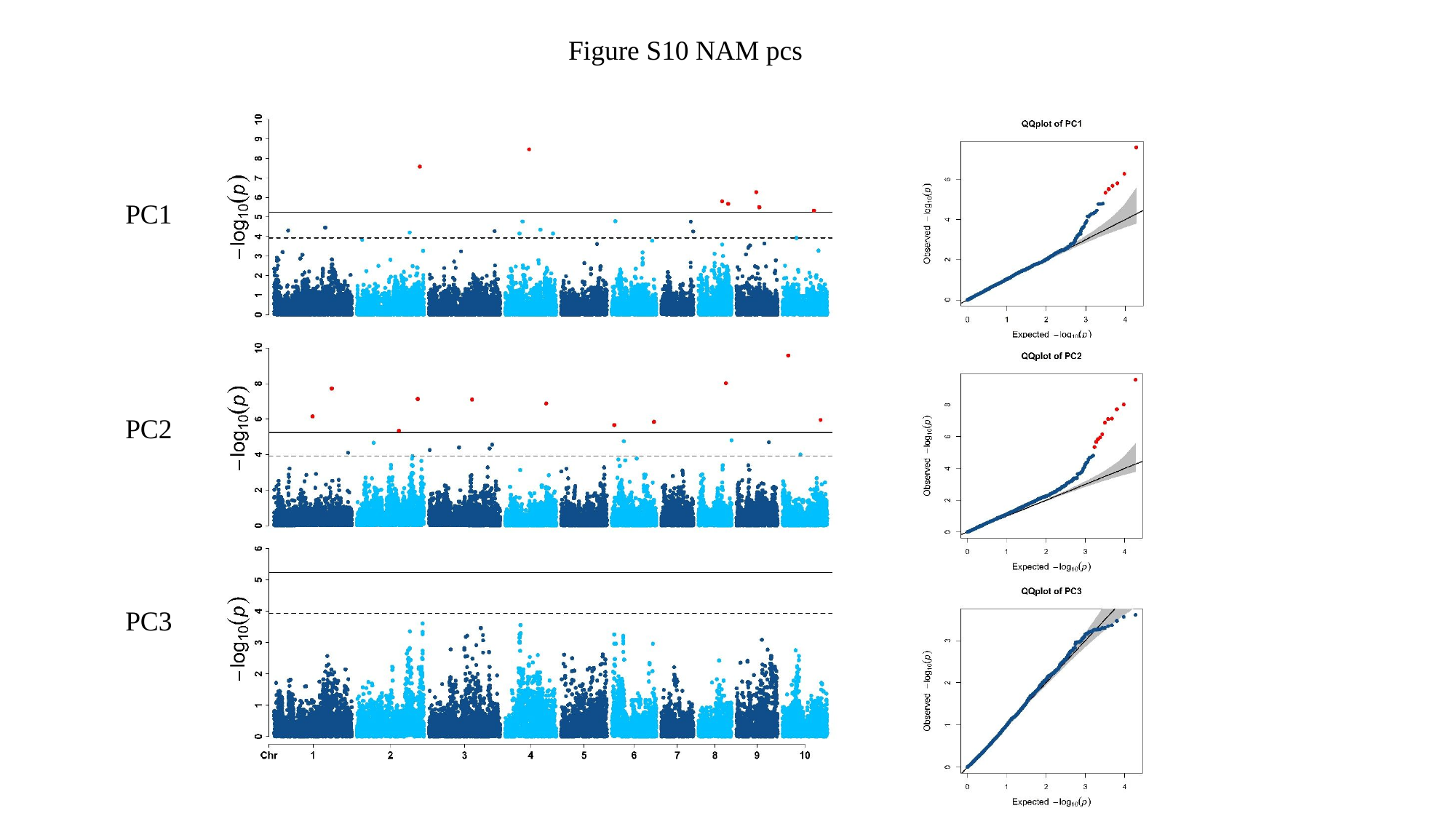

Figure S10 NAM pcs
PC1
PC2
PC3

## Slide 11
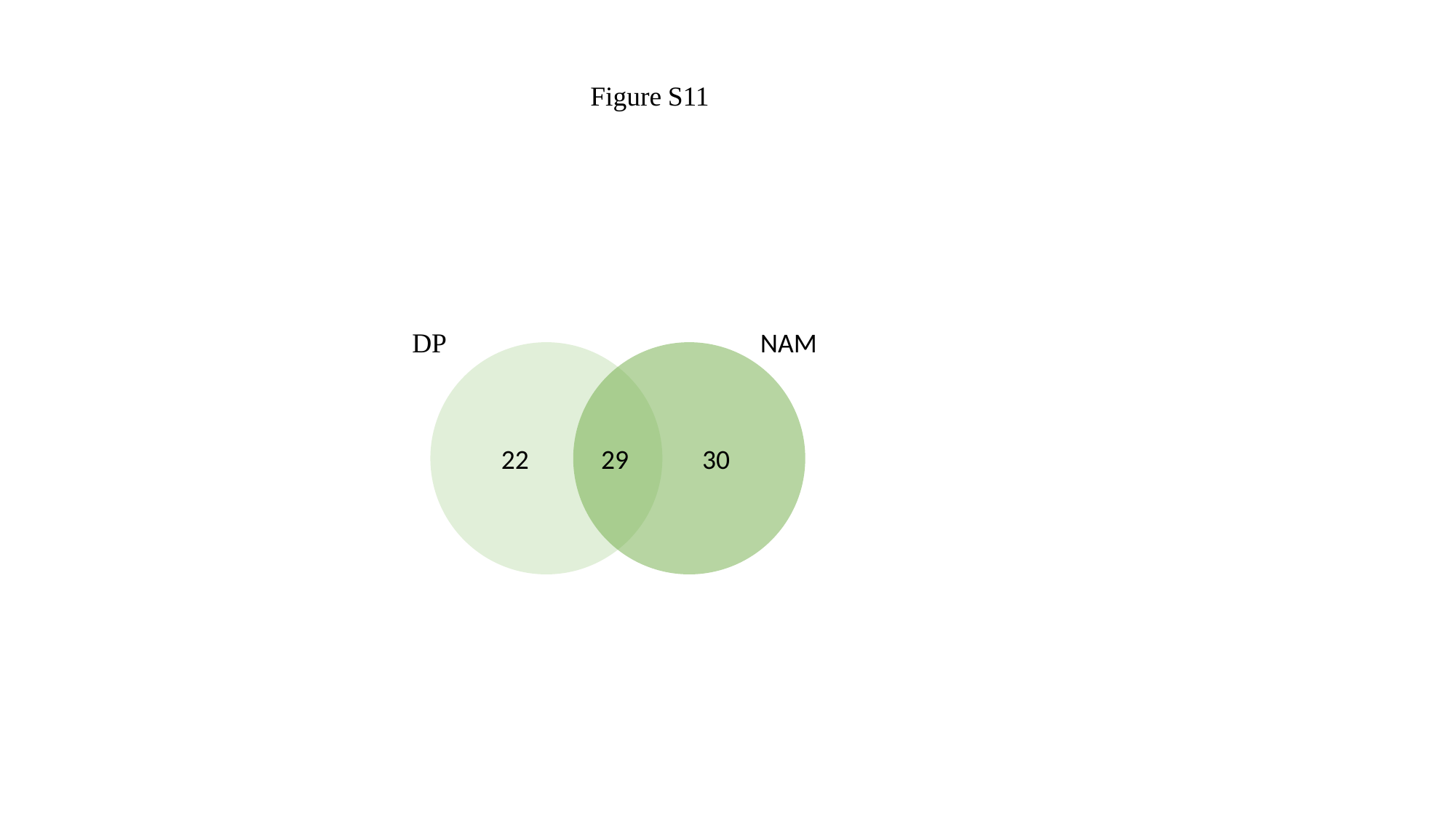

Figure S11
DP
NAM
22
29
30

## Slide 12
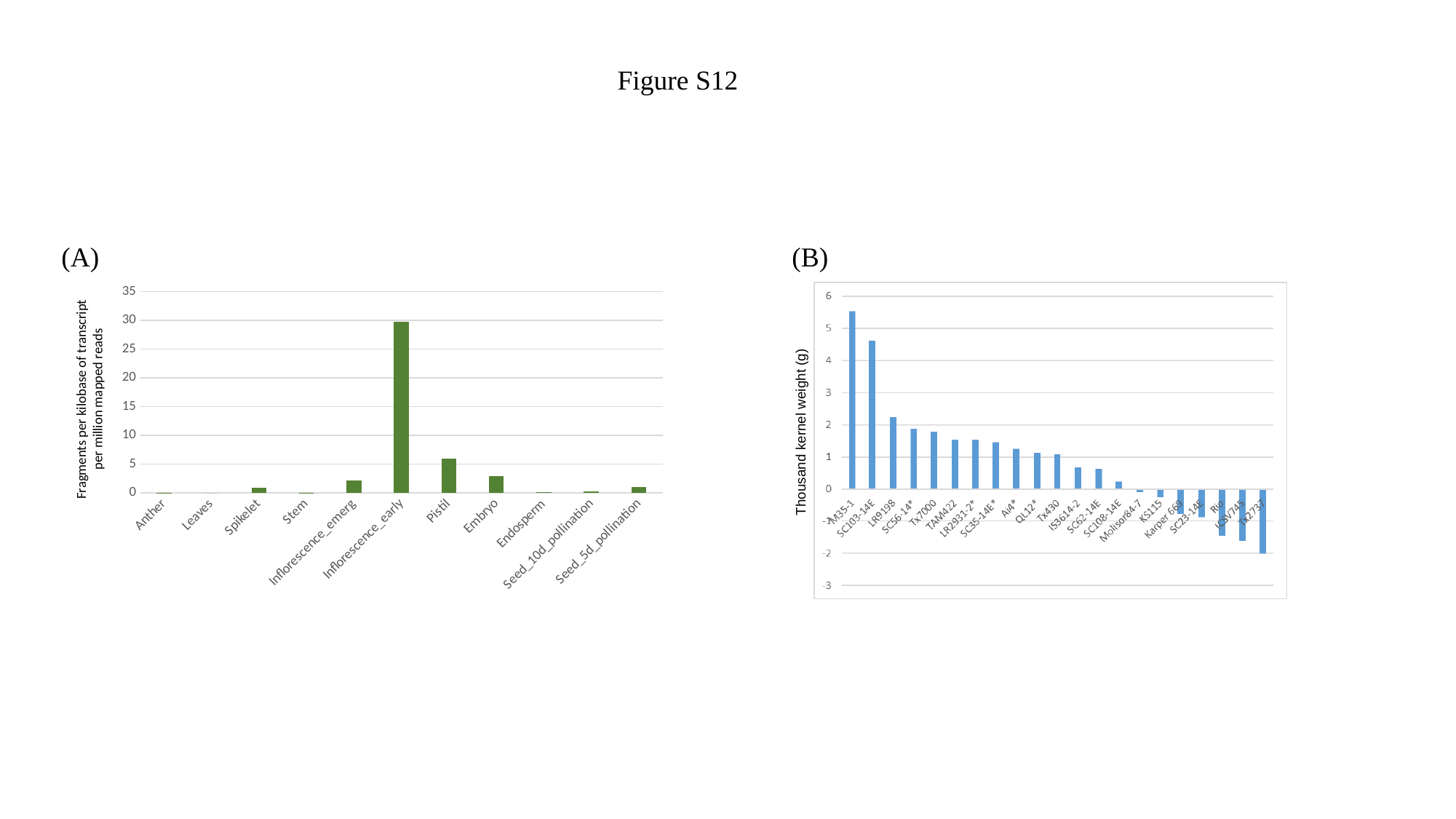

Figure S12
(A)
(B)
### Chart
| Category | |
|---|---|
| Anther | 0.0420066 |
| Leaves | 0.0 |
| Spikelet | 0.842635 |
| Stem | 0.0344034 |
| Inflorescence_emerg | 2.16953 |
| Inflorescence_early | 29.6973 |
| Pistil | 5.89623 |
| Embryo | 2.92481 |
| Endosperm | 0.126145 |
| Seed_10d_pollination | 0.246686 |
| Seed_5d_pollination | 1.06678 |
Fragments per kilobase of transcript per million mapped reads
Thousand kernel weight (g)
