# Supplementary material for: Large‐scale GWAS in sorghum reveals common genetic control of grain size among cereals
Source: Plant Biotechnol J. 2019 Nov 11;18(4):1093–105. doi: 10.1111/pbi.13284 (PMC7061873; doi:10.1111/pbi.13284)
Supplement: Supplementary file 4 — Table S3 Summary of field experiments. [file PBI-18-1093-s014.pdf]

Table S3 Summary of field experiments

Trial	Year	Location	Sowing date	Population	Number of plots	Number of genotypes
NAMGAT15	2014/2015	Gatton	22/10/2014	BC NAM	900	725
NAMHER15	2014/2015	Hermitage	9/10/2014	BC NAM	1508	1164
NAMGAT16	2015/2016	Gatton	24/09/2015	BC NAM	1521	1116
DPGAT16	2015/2016	Gatton	24/09/2015	DP	880	658
DPHER16	2015/2016	Hermitage	11/01/2016	DP	1400	888
