# Supplementary material for: Large‐scale GWAS in sorghum reveals common genetic control of grain size among cereals
Source: Plant Biotechnol J. 2019 Nov 11;18(4):1093–105. doi: 10.1111/pbi.13284 (PMC7061873; doi:10.1111/pbi.13284)
Supplement: Supplementary file 5 — Table S4 Racial group breakdown of the diversity panel. [file PBI-18-1093-s013.pdf]

Table S4 Racial group breakdown of the diversity panel

ID	Caudatum	Guinea	Kafir	Asian durras	E African durras	Group
B599	0.35	0.00	0.62	0.03	0.00	mixed
Early hegari	0.90	0.02	0.00	0.05	0.03	mixed
B010054	0.45	0.00	0.51	0.03	0.00	mixed
B35	0.06	0.01	0.24	0.53	0.15	mixed
B923296	0.41	0.00	0.52	0.05	0.02	mixed
B963676	0.42	0.00	0.45	0.09	0.03	mixed
B986604	0.43	0.02	0.54	0.01	0.01	mixed
BTx623	0.46	0.00	0.54	0.00	0.00	mixed
M35-1	0.86	0.01	0.12	0.01	0.00	mixed
RTAM422	0.27	0.00	0.49	0.21	0.03	mixed
RTx430	0.36	0.46	0.12	0.05	0.02	mixed
RTx7000	0.04	0.03	0.63	0.27	0.02	mixed
SC10-14E	0.00	0.00	0.04	0.37	0.59	mixed
SC100-14E	0.00	0.76	0.08	0.04	0.12	mixed
SC101-14E	0.12	0.12	0.69	0.03	0.04	mixed
SC1014-14E	0.04	0.00	0.08	0.86	0.02	mixed
SC1015-14E	0.12	0.02	0.14	0.57	0.15	mixed
SC1017-14E	0.07	0.05	0.08	0.65	0.15	mixed
SC1019-14E	0.76	0.04	0.04	0.12	0.04	mixed
SC1021-6	0.00	0.01	0.40	0.53	0.07	mixed
SC1022-14E	0.00	0.00	0.97	0.02	0.01	Kafir
SC1024-9	0.02	0.03	0.14	0.67	0.14	mixed
SC1025-14E	0.12	0.14	0.22	0.27	0.25	mixed
SC103-14E	0.81	0.03	0.10	0.04	0.02	mixed
SC1031-12E	0.07	0.04	0.19	0.48	0.22	mixed
SC1033-14E	0.07	0.03	0.14	0.62	0.14	mixed
SC1038-14E	0.94	0.02	0.00	0.02	0.02	mixed
SC1039-14E	0.00	0.00	1.00	0.00	0.00	Kafir
SC1040-14E	0.04	0.09	0.03	0.65	0.18	mixed
SC1046-14E	0.04	0.06	0.08	0.67	0.14	mixed
SC1049-14E	0.00	0.00	0.03	0.97	0.00	Asian durras
SC105-14E	0.07	0.01	0.03	0.89	0.01	mixed
SC1055-14E	0.94	0.02	0.04	0.00	0.00	mixed
SC1057-14E	0.92	0.01	0.06	0.01	0.00	mixed
SC106-14E	0.00	0.00	0.00	0.00	1.00	E African durras
SC1063-14E	0.08	0.48	0.41	0.00	0.03	mixed
SC1065-14E	0.91	0.02	0.05	0.00	0.02	mixed
SC1067-14E	0.54	0.25	0.02	0.15	0.04	mixed
SC1069-14E	0.97	0.01	0.01	0.01	0.00	Caudatum
SC1070-14E	0.86	0.04	0.01	0.08	0.00	mixed
SC1072-14	0.17	0.00	0.52	0.31	0.00	mixed
SC1074-12E	0.05	0.53	0.38	0.04	0.00	mixed
SC1076-12E	0.15	0.66	0.14	0.04	0.01	mixed
SC1077-14E	0.98	0.00	0.00	0.00	0.01	Caudatum

SC108-14E	1.00	0.00	0.00	0.00	0.00	Caudatum
SC1080-14E	0.00	0.00	1.00	0.00	0.00	Kafir
SC1082-6	0.30	0.32	0.24	0.04	0.10	mixed
SC1083-14E	0.06	0.51	0.25	0.00	0.18	mixed
SC1084-14E	0.06	0.47	0.31	0.00	0.16	mixed
SC1085-14E	0.03	0.02	0.09	0.84	0.02	mixed
SC1088-14E	0.15	0.08	0.03	0.73	0.02	mixed
SC109-14E	1.00	0.00	0.00	0.00	0.00	Caudatum
SC1097-3	0.00	0.00	0.62	0.38	0.00	mixed
SC110-14E	1.00	0.00	0.00	0.00	0.00	Caudatum
SC1101-14E	0.08	0.03	0.01	0.85	0.03	mixed
SC1103-14E	0.77	0.09	0.06	0.04	0.04	mixed
SC1104-14E	0.70	0.01	0.20	0.06	0.03	mixed
SC1107-9	0.08	0.02	0.44	0.38	0.07	mixed
SC1109-14E	0.15	0.20	0.58	0.00	0.07	mixed
SC111-14E	1.00	0.00	0.00	0.00	0.00	Caudatum
SC1111-14E	0.60	0.13	0.07	0.12	0.07	mixed
SC1114-6	0.35	0.16	0.35	0.14	0.01	mixed
SC1116-14E	0.46	0.06	0.42	0.06	0.01	mixed
SC1117-14E	0.40	0.33	0.11	0.13	0.04	mixed
SC1118-14E	0.71	0.03	0.17	0.06	0.03	mixed
SC112-14E	1.00	0.00	0.00	0.00	0.00	Caudatum
SC1123-14E	0.00	0.97	0.00	0.03	0.00	Guinea
SC1124-14E	0.00	1.00	0.00	0.00	0.00	Guinea
SC1125-14E	0.00	1.00	0.00	0.00	0.00	Guinea
SC113-14	0.76	0.02	0.16	0.03	0.03	mixed
SC1133-6	0.44	0.13	0.40	0.02	0.01	mixed
SC114-14E	0.92	0.03	0.01	0.03	0.01	mixed
SC115-14E	0.86	0.06	0.06	0.02	0.00	mixed
SC1154-14E	0.06	0.06	0.01	0.71	0.16	mixed
SC1155-14E	0.09	0.05	0.00	0.68	0.17	mixed
SC1158-5bk	0.01	0.11	0.66	0.14	0.09	mixed
SC1160-14E	0.08	0.00	0.13	0.01	0.79	mixed
SC1166-6	0.00	0.00	0.20	0.80	0.00	mixed
SC1170-6	0.02	0.03	0.31	0.48	0.17	mixed
SC1172-14E	0.45	0.01	0.46	0.08	0.00	mixed
SC1177-14E	0.00	0.01	0.04	0.00	0.95	E African durras
SC1178-9	0.59	0.16	0.19	0.06	0.01	mixed
SC1179-6	0.03	0.00	0.16	0.80	0.00	mixed
SC118-14E	0.88	0.02	0.06	0.04	0.00	mixed
SC1184-14E	0.03	0.05	0.11	0.45	0.36	mixed
SC1186-14E	0.11	0.11	0.09	0.59	0.10	mixed
SC119-14E	0.86	0.01	0.11	0.00	0.03	mixed
SC1193-3	0.00	0.01	0.47	0.48	0.03	mixed
SC12-14E	0.00	0.00	0.02	0.28	0.70	mixed
SC120-14E	0.98	0.00	0.00	0.02	0.00	Caudatum
SC1201-14E	0.19	0.11	0.58	0.08	0.03	mixed

SC1201-6-3	0.13	0.22	0.38	0.21	0.05	mixed
SC1203-14E	0.50	0.15	0.28	0.03	0.03	mixed
SC121-14E	0.05	0.02	0.93	0.00	0.00	mixed
SC1211-14E	0.36	0.17	0.36	0.08	0.02	mixed
SC1214-14E	0.75	0.03	0.12	0.08	0.02	mixed
SC1215-13E	0.15	0.01	0.58	0.22	0.03	mixed
SC1218	0.45	0.24	0.12	0.15	0.05	mixed
SC123-14E	0.00	0.06	0.16	0.44	0.34	mixed
SC124-14E	0.00	0.03	0.00	0.25	0.71	mixed
SC1261-14E	0.96	0.00	0.00	0.02	0.01	Caudatum
SC1262-14E	0.98	0.00	0.01	0.00	0.01	Caudatum
SC1263-14E	0.88	0.06	0.02	0.04	0.00	mixed
SC1271-13E	1.00	0.00	0.00	0.00	0.00	Caudatum
SC1272-5	0.27	0.00	0.19	0.53	0.01	mixed
SC1287-14E	0.00	0.01	0.21	0.00	0.78	mixed
SC1293-14E	0.47	0.35	0.09	0.07	0.02	mixed
SC13-14E	0.00	0.00	0.17	0.02	0.82	mixed
SC1300-14E	1.00	0.00	0.00	0.00	0.00	Caudatum
SC1302-14E	1.00	0.00	0.00	0.00	0.00	Caudatum
SC1305-14E	0.92	0.00	0.08	0.00	0.00	mixed
SC1307-14E	1.00	0.00	0.00	0.00	0.00	Caudatum
SC1313-14E	0.98	0.00	0.02	0.00	0.00	Caudatum
SC1314-14E	0.52	0.00	0.00	0.00	0.48	mixed
SC1316-14E	0.81	0.00	0.19	0.00	0.00	mixed
SC1317-14E	0.98	0.00	0.00	0.02	0.00	Caudatum
SC1318-14E	1.00	0.00	0.00	0.00	0.00	Caudatum
SC1319-14E	1.00	0.00	0.00	0.00	0.00	Caudatum
SC132-12E	0.13	0.04	0.00	0.67	0.17	mixed
SC1320-14E	0.96	0.02	0.02	0.00	0.00	Caudatum
SC1321-14E	0.99	0.00	0.00	0.01	0.00	Caudatum
SC1322-14E	0.21	0.12	0.07	0.43	0.16	mixed
SC1325-14E	0.33	0.13	0.03	0.38	0.13	mixed
SC1328-14E	0.62	0.07	0.13	0.12	0.06	mixed
SC1329-14E	0.23	0.12	0.11	0.40	0.15	mixed
SC1330-14E	0.25	0.16	0.02	0.41	0.15	mixed
SC1332-14E	0.12	0.74	0.03	0.11	0.00	mixed
SC1333-14E	0.13	0.69	0.11	0.07	0.00	mixed
SC1334-5	0.11	0.83	0.00	0.04	0.01	mixed
SC1337-14E	0.07	0.62	0.13	0.09	0.09	mixed
SC1339-14E	0.08	0.82	0.06	0.04	0.00	mixed
SC134-14E	0.02	0.09	0.06	0.59	0.25	mixed
SC1341-14E	0.08	0.83	0.06	0.03	0.01	mixed
SC1342-14E	0.08	0.84	0.04	0.02	0.01	mixed
SC1345-14E	0.92	0.02	0.01	0.03	0.02	mixed
SC135-14E	0.09	0.07	0.03	0.48	0.32	mixed
SC1351-14E	0.64	0.11	0.04	0.14	0.06	mixed
SC1356-14E	0.62	0.09	0.13	0.11	0.06	mixed

SC137-14E	0.00	0.00	0.00	0.00	1.00	E African durras
SC139-14E	0.04	0.01	0.09	0.36	0.49	mixed
SC140-14E	0.05	0.03	0.02	0.35	0.54	mixed
SC141-14E	0.03	0.05	0.01	0.39	0.51	mixed
SC142-14E	0.19	0.07	0.28	0.38	0.09	mixed
SC1424-2	0.01	0.03	0.28	0.52	0.15	mixed
SC1426-10E	0.09	0.85	0.03	0.03	0.00	mixed
SC1440-8	0.06	0.39	0.21	0.01	0.32	mixed
SC145-14E	0.00	0.00	0.04	0.00	0.96	E African durras
SC146-14E	0.89	0.00	0.07	0.00	0.03	mixed
SC147-14E	0.01	0.01	0.05	0.00	0.93	mixed
SC1476-8	0.65	0.09	0.11	0.10	0.05	mixed
SC15-14E	0.13	0.05	0.04	0.00	0.78	mixed
SC150-14E	0.01	0.02	0.03	0.35	0.59	mixed
SC154-14E	0.01	0.01	0.05	0.13	0.80	mixed
SC155-14E	0.02	0.01	0.02	0.36	0.59	mixed
SC156-14E	0.00	0.00	0.00	0.00	1.00	E African durras
SC157-6	0.00	0.12	0.82	0.06	0.00	mixed
SC158-14E	0.00	0.00	0.00	0.47	0.53	mixed
SC159-11E	0.00	0.02	0.04	0.28	0.66	mixed
SC16-14E	0.05	0.02	0.11	0.08	0.74	mixed
SC161-14E	0.00	0.01	0.11	0.44	0.44	mixed
SC165-14E	0.65	0.07	0.12	0.08	0.09	mixed
SC166-14E	0.00	0.00	0.00	0.00	1.00	E African durras
SC167-14E	0.07	0.00	0.06	0.00	0.87	mixed
SC17-14E	0.03	0.04	0.12	0.08	0.74	mixed
SC170-14E	0.94	0.00	0.06	0.00	0.00	mixed
SC170-6-17	0.84	0.01	0.13	0.01	0.01	mixed
SC170-6-8	0.51	0.33	0.07	0.06	0.02	mixed
SC171-14E	0.56	0.03	0.38	0.03	0.00	mixed
SC172-12E	1.00	0.00	0.00	0.00	0.00	Caudatum
SC173-14E	1.00	0.00	0.00	0.00	0.00	Caudatum
SC175-14E	0.96	0.00	0.00	0.04	0.00	Caudatum
SC176-14E	0.88	0.00	0.12	0.00	0.00	mixed
SC180-14E	0.00	0.00	0.04	0.44	0.52	mixed
SC182-14E	0.00	0.00	0.00	0.26	0.74	mixed
SC183-14E	0.34	0.17	0.40	0.03	0.05	mixed
SC184-14E	0.90	0.00	0.10	0.00	0.00	mixed
SC185-14E	0.80	0.06	0.11	0.01	0.01	mixed
SC186-14E	0.16	0.68	0.03	0.12	0.01	mixed
SC187-14E	0.15	0.61	0.06	0.14	0.05	mixed
SC188-14E	0.19	0.57	0.09	0.13	0.02	mixed
SC189-2	0.01	0.00	0.01	0.98	0.00	Asian durras
SC19-14E	0.00	0.00	0.00	0.00	1.00	E African durras
SC190-6	0.00	0.00	0.29	0.71	0.00	mixed
SC191-14E	0.10	0.05	0.13	0.72	0.00	mixed
SC192-14E	0.03	0.05	0.11	0.80	0.00	mixed

SC193-14E	0.08	0.78	0.03	0.10	0.00	mixed
SC195-14E	0.10	0.18	0.49	0.17	0.06	mixed
SC196-14E	0.04	0.03	0.03	0.88	0.03	mixed
SC2-14E	0.00	0.00	0.00	0.07	0.93	mixed
SC20-14E	0.00	0.00	0.00	0.00	1.00	E African durras
SC200-14E	0.00	0.00	0.00	1.00	0.00	Asian durras
SC201-14E	0.02	0.01	0.03	0.94	0.00	mixed
SC202-14E	0.00	0.00	0.04	0.96	0.00	Asian durras
SC203-14E	0.00	0.00	0.01	0.99	0.00	Asian durras
SC206-14E	0.00	0.00	0.00	1.00	0.00	Asian durras
SC207-14E	0.00	0.00	0.00	1.00	0.00	Asian durras
SC208-14E	0.00	0.03	0.00	0.97	0.00	Asian durras
SC209-14E	0.06	0.02	0.00	0.92	0.00	mixed
SC21-14E	0.00	0.00	0.01	0.00	0.99	E African durras
SC210-14E	0.00	0.03	0.00	0.97	0.00	Asian durras
SC211-14E	0.00	0.01	0.00	0.99	0.00	Asian durras
SC212-14E	0.00	0.01	0.00	0.99	0.00	Asian durras
SC213-14E	0.00	0.53	0.00	0.47	0.00	mixed
SC214-14E	0.01	0.01	0.12	0.86	0.00	mixed
SC215-14E	0.00	0.01	0.00	0.99	0.00	Asian durras
SC216-14E	0.05	0.01	0.07	0.85	0.02	mixed
SC217-14E	0.00	0.04	0.00	0.96	0.00	Asian durras
SC218-14E	0.04	0.01	0.00	0.94	0.01	mixed
SC22-14E	0.06	0.06	0.10	0.61	0.17	mixed
SC220-14E	0.02	0.01	0.00	0.96	0.00	Asian durras
SC221-14E	0.00	0.02	0.00	0.98	0.00	Asian durras
SC223-14E	0.77	0.05	0.10	0.02	0.05	mixed
SC224-14E	0.00	0.00	0.01	0.00	0.99	E African durras
SC23-14E	0.03	0.06	0.12	0.63	0.17	mixed
SC230-14E	0.03	0.08	0.00	0.90	0.00	mixed
SC231-14E	0.06	0.03	0.00	0.91	0.00	mixed
SC233-14E	0.00	0.00	0.00	1.00	0.00	Asian durras
SC235-14E	0.06	0.09	0.36	0.14	0.34	mixed
SC236-3	0.00	0.00	0.46	0.51	0.03	mixed
SC24-14E	0.03	0.05	0.16	0.61	0.16	mixed
SC240-14E	0.00	0.00	0.00	1.00	0.00	Asian durras
SC241-14E	0.08	0.12	0.40	0.37	0.03	mixed
SC242-14E	0.12	0.17	0.55	0.11	0.05	mixed
SC243-14E	0.09	0.16	0.60	0.10	0.05	mixed
SC244-14E	0.14	0.11	0.55	0.15	0.05	mixed
SC245-14E	0.05	0.09	0.07	0.79	0.00	mixed
SC247-14E	0.09	0.15	0.53	0.20	0.04	mixed
SC248-14E	0.07	0.14	0.48	0.26	0.04	mixed
SC249-14E	0.00	0.67	0.30	0.00	0.03	mixed
SC25-14E	0.06	0.04	0.11	0.64	0.15	mixed
SC250-14E	0.11	0.19	0.49	0.16	0.04	mixed
SC252-14E	0.07	0.18	0.57	0.15	0.03	mixed

SC254-14E	0.11	0.15	0.47	0.24	0.03	mixed
SC256-14E	0.00	1.00	0.00	0.00	0.00	Guinea
SC257-14E	0.47	0.07	0.35	0.11	0.00	mixed
SC258-14E	0.14	0.16	0.63	0.00	0.07	mixed
SC259-14E	0.96	0.00	0.04	0.00	0.00	Caudatum
SC260-6	0.07	0.56	0.30	0.07	0.00	mixed
SC261-14E	0.24	0.66	0.06	0.04	0.00	mixed
SC262-14E	0.32	0.54	0.05	0.05	0.04	mixed
SC265-14E	0.05	0.67	0.22	0.03	0.03	mixed
SC266-6	0.08	0.57	0.28	0.08	0.00	mixed
SC267-14E	0.27	0.49	0.16	0.06	0.02	mixed
SC268-14E	0.63	0.10	0.12	0.13	0.03	mixed
SC269-14E	0.30	0.06	0.54	0.10	0.00	mixed
SC27-14E	0.02	0.04	0.02	0.44	0.48	mixed
SC270-14E	0.02	0.95	0.00	0.03	0.00	mixed
SC271-14E	0.00	0.95	0.05	0.00	0.00	Guinea
SC272-14E	0.00	0.97	0.03	0.00	0.00	Guinea
SC273-14E	0.08	0.84	0.05	0.03	0.00	mixed
SC276-14E	0.32	0.39	0.22	0.07	0.00	mixed
SC277-14E	0.00	0.99	0.01	0.00	0.00	Guinea
SC278-14E	0.11	0.70	0.13	0.02	0.04	mixed
SC279-14E	0.00	1.00	0.00	0.00	0.00	Guinea
SC28-14E	0.05	0.06	0.08	0.64	0.17	mixed
SC280-14E	0.00	0.88	0.12	0.00	0.00	mixed
SC281-14E	0.10	0.42	0.32	0.00	0.15	mixed
SC283-14E	0.08	0.31	0.56	0.00	0.05	mixed
SC284-14E	0.59	0.39	0.01	0.00	0.00	mixed
SC285-14E	0.00	1.00	0.00	0.00	0.00	Guinea
SC287-14E	0.00	1.00	0.00	0.00	0.00	Guinea
SC289-14E	0.00	0.95	0.02	0.02	0.00	Guinea
SC29-14E	0.04	0.07	0.12	0.65	0.13	mixed
SC290-14E	0.00	1.00	0.00	0.00	0.00	Guinea
SC291-14E	0.00	1.00	0.00	0.00	0.00	Guinea
SC293-14E	0.02	0.96	0.00	0.02	0.00	Guinea
SC295-11E	0.00	1.00	0.00	0.00	0.00	Guinea
SC296-14E	0.09	0.84	0.07	0.00	0.00	mixed
SC297-14E	0.00	0.99	0.01	0.00	0.00	Guinea
SC299-14E	0.01	0.99	0.00	0.00	0.00	Guinea
SC3-3	0.00	0.01	0.48	0.40	0.11	mixed
SC30-14E	0.01	0.01	0.09	0.20	0.68	mixed
SC300-14E	0.13	0.87	0.00	0.00	0.00	mixed
SC301-14E	0.03	0.92	0.04	0.00	0.00	mixed
SC305-14E	0.05	0.45	0.33	0.00	0.17	mixed
SC306-14E	0.00	0.00	0.00	0.00	1.00	E African durras
SC307-14E	0.00	0.00	0.00	1.00	0.00	Asian durras
SC311-14E	0.26	0.35	0.12	0.17	0.10	mixed
SC314-6	0.09	0.08	0.79	0.00	0.03	mixed

SC319-14E	0.31	0.25	0.16	0.15	0.14	mixed
SC320-14E	0.56	0.20	0.04	0.14	0.05	mixed
SC323-14E	0.39	0.25	0.25	0.05	0.07	mixed
SC324-14	0.87	0.02	0.11	0.00	0.00	mixed
SC325-14E	0.28	0.26	0.23	0.05	0.18	mixed
SC326-6	0.58	0.00	0.35	0.07	0.00	mixed
SC328-14E	0.97	0.00	0.03	0.00	0.00	Caudatum
SC329-14E	0.51	0.28	0.14	0.07	0.00	mixed
SC330-14E	0.90	0.01	0.07	0.01	0.00	mixed
SC331-14E	0.04	0.45	0.46	0.04	0.01	mixed
SC333-14E	0.64	0.18	0.02	0.11	0.04	mixed
SC334-14E	0.75	0.09	0.04	0.09	0.03	mixed
SC335-14E	0.49	0.19	0.10	0.19	0.04	mixed
SC336-14E	0.66	0.15	0.00	0.13	0.07	mixed
SC338-14E	0.57	0.11	0.04	0.20	0.08	mixed
SC339-14E	0.44	0.22	0.08	0.18	0.08	mixed
SC340-14E	0.51	0.16	0.04	0.22	0.08	mixed
SC342-14E	0.32	0.55	0.07	0.06	0.01	mixed
SC343-9	0.54	0.04	0.23	0.15	0.03	mixed
SC344-14E	0.09	0.25	0.46	0.14	0.07	mixed
SC345-14E	0.32	0.25	0.19	0.20	0.04	mixed
SC346-14E	0.52	0.17	0.09	0.18	0.03	mixed
SC347-14E	0.19	0.54	0.07	0.17	0.04	mixed
SC348-14E	0.06	0.79	0.06	0.09	0.00	mixed
SC349-14E	0.36	0.25	0.13	0.21	0.05	mixed
SC35-14E	0.05	0.05	0.09	0.64	0.17	mixed
SC350-14E	0.96	0.00	0.04	0.00	0.00	Caudatum
SC351-14E	0.01	0.92	0.03	0.00	0.04	mixed
SC352-14E	0.57	0.10	0.03	0.20	0.10	mixed
SC353-14E	0.89	0.07	0.00	0.01	0.04	mixed
SC354-14E	0.01	0.91	0.06	0.00	0.02	mixed
SC356-14E	0.27	0.66	0.03	0.04	0.00	mixed
SC358-14E	0.05	0.80	0.04	0.11	0.00	mixed
SC36-14E	0.03	0.06	0.13	0.64	0.14	mixed
SC362-14E	0.10	0.78	0.00	0.10	0.02	mixed
SC366-14E	0.04	0.85	0.09	0.03	0.00	mixed
SC367-14E	0.08	0.81	0.03	0.08	0.00	mixed
SC368-14E	0.08	0.77	0.05	0.10	0.01	mixed
SC369-14E	0.07	0.76	0.06	0.09	0.02	mixed
SC37-14E	0.00	0.00	0.00	0.11	0.89	mixed
SC370-14E	0.09	0.74	0.07	0.11	0.00	mixed
SC371-14E	0.34	0.51	0.08	0.07	0.00	mixed
SC372-14E	0.03	0.80	0.13	0.04	0.00	mixed
SC373-14E	0.16	0.76	0.01	0.07	0.00	mixed
SC374-14E	0.03	0.96	0.00	0.00	0.00	Guinea
SC377-14E	0.00	0.98	0.02	0.00	0.00	Guinea
SC38-14E	0.05	0.07	0.02	0.67	0.20	mixed

SC380-14E	0.36	0.35	0.28	0.01	0.00	mixed
SC386-14E	0.00	0.79	0.18	0.02	0.00	mixed
SC387-14E	0.01	0.78	0.02	0.00	0.18	mixed
SC389-14E	0.00	1.00	0.00	0.00	0.00	Guinea
SC391-14E	0.08	0.81	0.01	0.09	0.00	mixed
SC392-14E	0.06	0.81	0.03	0.08	0.01	mixed
SC396-14E	0.09	0.79	0.01	0.10	0.01	mixed
SC397-14E	0.14	0.63	0.12	0.10	0.02	mixed
SC398-14E	0.31	0.00	0.69	0.00	0.00	mixed
SC399-14E	0.16	0.66	0.06	0.11	0.01	mixed
SC402-14E	0.03	0.84	0.05	0.07	0.00	mixed
SC403-14E	0.00	1.00	0.00	0.00	0.00	Guinea
SC405-14E	0.90	0.04	0.00	0.03	0.02	mixed
SC406-14E	0.23	0.68	0.01	0.06	0.02	mixed
SC407-14E	0.33	0.44	0.07	0.08	0.08	mixed
SC408-14E	0.09	0.86	0.05	0.00	0.00	mixed
SC409-14E	0.20	0.57	0.08	0.14	0.00	mixed
SC41-5	0.09	0.08	0.31	0.42	0.11	mixed
SC410-14E	0.00	0.00	0.00	1.00	0.00	Asian durras
SC410-6	0.00	0.67	0.27	0.06	0.00	mixed
SC411-14E	0.52	0.12	0.17	0.10	0.09	mixed
SC417-14E	0.12	0.12	0.37	0.31	0.08	mixed
SC418-14E	0.71	0.12	0.12	0.02	0.04	mixed
SC42-14E	0.26	0.23	0.23	0.02	0.26	mixed
SC420-14E	0.76	0.06	0.06	0.08	0.04	mixed
SC422-14E	0.49	0.19	0.23	0.04	0.06	mixed
SC423-14	0.95	0.00	0.05	0.00	0.00	Caudatum
SC424-14E	0.93	0.00	0.07	0.00	0.00	mixed
SC425-14E	0.55	0.20	0.05	0.16	0.04	mixed
SC43-14E	0.00	0.00	0.00	0.00	1.00	E African durras
SC430-14E	0.08	0.06	0.00	0.69	0.17	mixed
SC435-14E	0.00	0.00	0.96	0.04	0.00	Kafir
SC436-9	0.01	0.02	0.11	0.87	0.00	mixed
SC437-14E	0.02	0.03	0.06	0.89	0.00	mixed
SC438-3	0.00	0.00	0.64	0.32	0.03	mixed
SC44-14E	0.00	0.00	0.00	0.00	1.00	E African durras
SC441-14E	0.00	0.10	0.03	0.88	0.00	mixed
SC442-14E	0.00	0.04	0.03	0.93	0.00	mixed
SC445-14E	0.10	0.15	0.44	0.28	0.03	mixed
SC449-14E	0.10	0.12	0.43	0.32	0.04	mixed
SC450-14E	0.02	0.03	0.06	0.84	0.05	mixed
SC451-14E	0.00	0.06	0.22	0.72	0.00	mixed
SC452-14E	0.00	0.00	0.00	1.00	0.00	Asian durras
SC454-14E	0.07	0.04	0.47	0.32	0.10	mixed
SC456-6	0.01	0.00	0.18	0.81	0.00	mixed
SC457-14E	0.03	0.00	0.14	0.82	0.01	mixed
SC458-12E	0.00	0.03	0.19	0.78	0.00	mixed

SC460-14E	0.03	0.00	0.05	0.91	0.01	mixed
SC462-14E	0.00	0.02	0.01	0.96	0.00	Asian durras
SC464-14E	0.02	0.03	0.08	0.88	0.00	mixed
SC465-14E	0.18	0.28	0.40	0.07	0.07	mixed
SC466-14E	0.11	0.01	0.71	0.14	0.03	mixed
SC467-14E	0.00	0.00	0.25	0.75	0.00	mixed
SC468-14E	0.00	0.00	0.42	0.58	0.00	mixed
SC469-14E	0.01	0.00	0.01	0.98	0.00	Asian durras
SC470-6	0.00	0.00	0.36	0.64	0.00	mixed
SC471-14E	0.00	0.00	0.00	1.00	0.00	Asian durras
SC472-14E	0.01	0.00	0.00	0.99	0.00	Asian durras
SC473-14E	0.02	0.00	0.07	0.91	0.00	mixed
SC475-14E	0.00	0.00	0.01	0.99	0.00	Asian durras
SC477-14E	0.00	0.00	0.06	0.94	0.00	mixed
SC479-14E	0.00	0.00	0.00	1.00	0.00	Asian durras
SC48-14E	0.44	0.18	0.13	0.19	0.06	mixed
SC480-14E	0.00	0.00	0.00	1.00	0.00	Asian durras
SC482-14E	0.00	0.00	0.00	1.00	0.00	Asian durras
SC483-14E	0.00	0.00	0.00	1.00	0.00	Asian durras
SC484-14E	0.00	0.00	0.00	0.87	0.13	mixed
SC485-14E	0.00	0.00	0.00	1.00	0.00	Asian durras
SC489-14E	0.00	0.00	0.00	1.00	0.00	Asian durras
SC490-14E	0.00	0.00	0.00	0.87	0.13	mixed
SC492-14E	0.00	0.00	0.00	0.99	0.00	Asian durras
SC493-14E	0.00	0.00	0.98	0.02	0.00	Kafir
SC494-14E	0.00	0.00	0.04	0.96	0.00	Asian durras
SC497-14E	0.00	0.00	0.03	0.97	0.00	Asian durras
SC498-14E	0.00	0.00	0.00	1.00	0.00	Asian durras
SC499-14E	0.00	0.00	0.00	1.00	0.00	Asian durras
SC500-9	0.00	0.00	0.02	0.98	0.00	Asian durras
SC501-14E	0.51	0.08	0.00	0.37	0.05	mixed
SC502-14E	0.51	0.10	0.14	0.20	0.05	mixed
SC505-14E	0.97	0.00	0.00	0.00	0.03	Caudatum
SC51-14E	0.47	0.17	0.09	0.23	0.05	mixed
SC512-14E	0.08	0.06	0.38	0.45	0.03	mixed
SC514-14E	0.06	0.15	0.47	0.29	0.02	mixed
SC515-14E	0.00	1.00	0.00	0.00	0.00	Guinea
SC516-14E	0.09	0.15	0.46	0.25	0.04	mixed
SC517-9	0.04	0.40	0.37	0.13	0.06	mixed
SC519-14E	0.00	1.00	0.00	0.00	0.00	Guinea
SC520-14E	0.00	0.96	0.04	0.00	0.00	Guinea
SC521-6	0.00	0.81	0.18	0.01	0.00	mixed
SC523-14E	0.00	1.00	0.00	0.00	0.00	Guinea
SC525-9	0.00	0.89	0.11	0.00	0.00	mixed
SC526-14E	0.00	0.96	0.04	0.00	0.00	Guinea
SC527-14E	0.00	0.92	0.06	0.02	0.00	mixed
SC529-14E	0.00	0.95	0.05	0.00	0.00	Guinea

SC532-14E	0.00	1.00	0.00	0.00	0.00	Guinea
SC536-14E	0.00	1.00	0.00	0.00	0.00	Guinea
SC537-14E	0.00	0.93	0.03	0.02	0.02	mixed
SC538-14E	0.09	0.65	0.16	0.10	0.00	mixed
SC54-14E	0.50	0.13	0.07	0.22	0.08	mixed
SC540-6	0.00	0.80	0.20	0.00	0.00	mixed
SC544-14E	0.00	0.97	0.03	0.00	0.00	Guinea
SC545-14E	0.00	0.96	0.02	0.01	0.00	Guinea
SC546-14E	0.00	0.95	0.05	0.00	0.00	mixed
SC547-6	0.00	0.94	0.06	0.00	0.00	mixed
SC55-14E	0.53	0.12	0.05	0.25	0.05	mixed
SC550-14E	0.00	0.44	0.21	0.02	0.33	mixed
SC553-14E	0.00	0.99	0.00	0.01	0.00	Guinea
SC556-3	0.00	0.00	0.97	0.03	0.00	Kafir
SC557-14E	0.14	0.26	0.50	0.00	0.10	mixed
SC558-14E	0.21	0.22	0.44	0.05	0.09	mixed
SC56-14E	0.63	0.06	0.11	0.15	0.04	mixed
SC562-14E	0.68	0.13	0.03	0.10	0.05	mixed
SC563-14E	0.47	0.18	0.19	0.14	0.03	mixed
SC565-14E	0.20	0.49	0.23	0.05	0.04	mixed
SC566-14E	0.12	0.84	0.02	0.03	0.00	mixed
SC567-14E	0.10	0.85	0.03	0.02	0.01	mixed
SC57-14E	0.98	0.00	0.01	0.00	0.01	Caudatum
SC572-14E	0.69	0.04	0.05	0.17	0.05	mixed
SC574-14E	0.85	0.05	0.09	0.02	0.00	mixed
SC575-14E	0.60	0.14	0.02	0.19	0.04	mixed
SC578-14E	0.13	0.19	0.61	0.00	0.07	mixed
SC58-14E	0.64	0.08	0.09	0.13	0.07	mixed
SC580-14E	0.00	0.00	0.02	0.98	0.00	Asian durras
SC582-14E	0.05	0.09	0.41	0.43	0.02	mixed
SC586-14E	0.81	0.00	0.17	0.01	0.00	mixed
SC589-14E	0.00	0.00	0.00	1.00	0.00	Asian durras
SC59-12E	0.31	0.22	0.32	0.08	0.07	mixed
SC590-14E	0.32	0.20	0.26	0.16	0.07	mixed
SC593-14E	0.38	0.27	0.18	0.08	0.09	mixed
SC598-14E	0.00	0.02	0.07	0.26	0.64	mixed
SC599-11E	0.59	0.00	0.41	0.00	0.00	mixed
SC6-14E	0.42	0.17	0.25	0.00	0.16	mixed
SC60-14E	0.97	0.01	0.00	0.02	0.00	Caudatum
SC600-14E	0.06	0.06	0.07	0.65	0.16	mixed
SC601-14E	0.46	0.11	0.42	0.00	0.01	mixed
SC602-6	0.13	0.00	0.27	0.00	0.60	mixed
SC603-14E	0.00	0.00	0.95	0.04	0.00	Kafir
SC604-6	0.30	0.13	0.44	0.10	0.02	mixed
SC605-14E	0.04	0.41	0.19	0.05	0.32	mixed
SC606-14E	0.46	0.02	0.36	0.10	0.06	mixed
SC609-14E	0.85	0.05	0.02	0.04	0.03	mixed

SC61-14E	0.41	0.27	0.14	0.10	0.08	mixed
SC614-14E	0.00	0.00	0.85	0.13	0.02	mixed
SC615-9	0.01	0.03	0.58	0.38	0.00	mixed
SC618-6	0.00	0.00	0.22	0.78	0.00	mixed
SC62-14E	0.38	0.21	0.17	0.17	0.07	mixed
SC620-14E	0.06	0.17	0.50	0.25	0.02	mixed
SC621-14E	0.02	0.00	0.24	0.71	0.03	mixed
SC623-14E	0.14	0.15	0.50	0.15	0.05	mixed
SC624-14E	0.17	0.10	0.69	0.03	0.00	mixed
SC625-14E	0.00	0.00	0.88	0.12	0.00	mixed
SC626-14E	0.00	0.02	0.94	0.04	0.00	mixed
SC627-14E	0.00	0.00	0.93	0.07	0.00	mixed
SC628-14E	0.00	0.00	1.00	0.00	0.00	Kafir
SC629-14E	0.00	0.00	0.99	0.00	0.00	Kafir
SC63-14E	0.59	0.11	0.12	0.12	0.06	mixed
SC630-14E	0.00	0.00	1.00	0.00	0.00	Kafir
SC631-6	0.00	0.00	1.00	0.00	0.00	Kafir
SC632-14	0.00	0.00	1.00	0.00	0.00	Kafir
SC634-14E	0.00	0.00	0.89	0.10	0.01	mixed
SC635-14E	0.83	0.08	0.00	0.07	0.02	mixed
SC636-6	0.74	0.00	0.26	0.00	0.00	mixed
SC637-14E	0.85	0.00	0.14	0.02	0.00	mixed
SC639-6	0.61	0.00	0.36	0.03	0.00	mixed
SC64-14E	0.89	0.00	0.11	0.00	0.00	mixed
SC641-14E	0.91	0.00	0.09	0.00	0.00	mixed
SC642-14E	0.91	0.00	0.09	0.00	0.00	mixed
SC643-14E	0.88	0.00	0.12	0.00	0.00	mixed
SC644-14E	0.94	0.00	0.06	0.00	0.00	mixed
SC645-14E	0.82	0.00	0.17	0.01	0.00	mixed
SC646-14E	0.04	0.00	0.96	0.00	0.00	Kafir
SC647-14E	0.02	0.00	0.98	0.00	0.00	Kafir
SC648-14E	0.00	0.00	1.00	0.00	0.00	Kafir
SC649-14E	0.66	0.09	0.23	0.00	0.03	mixed
SC650-14E	0.00	0.02	0.00	0.98	0.00	Asian durras
SC652-9	0.00	0.00	1.00	0.00	0.00	Kafir
SC653-14E	0.00	0.00	1.00	0.00	0.00	Kafir
SC654-14E	0.00	0.00	1.00	0.00	0.00	Kafir
SC655-14E	0.83	0.06	0.07	0.00	0.04	mixed
SC657-14E	0.00	0.00	1.00	0.00	0.00	Kafir
SC659-14E	0.35	0.03	0.47	0.13	0.02	mixed
SC66-14E	0.36	0.23	0.29	0.05	0.07	mixed
SC663-14E	0.00	0.00	1.00	0.00	0.00	Kafir
SC67-14E	0.35	0.25	0.22	0.10	0.08	mixed
SC671-14E	0.11	0.16	0.69	0.00	0.03	mixed
SC672-14E	0.03	0.00	0.97	0.00	0.00	Kafir
SC673-14E	0.00	0.00	1.00	0.00	0.00	Kafir
SC679-14E	0.49	0.16	0.29	0.03	0.03	mixed

SC680-14E	0.00	0.01	0.91	0.07	0.01	mixed
SC681-14E	0.93	0.00	0.07	0.00	0.00	mixed
SC686-14E	0.96	0.00	0.04	0.00	0.00	Caudatum
SC687-14E	0.94	0.02	0.02	0.01	0.01	mixed
SC69-14E	0.38	0.19	0.32	0.07	0.04	mixed
SC690-14E	0.83	0.00	0.17	0.00	0.00	mixed
SC691-14E	0.71	0.07	0.12	0.07	0.04	mixed
SC692-14E	0.92	0.00	0.08	0.00	0.00	mixed
SC693-14E	0.91	0.00	0.09	0.00	0.00	mixed
SC694-14E	0.86	0.03	0.08	0.03	0.00	mixed
SC695-9	0.87	0.00	0.13	0.00	0.00	mixed
SC7-14E	0.00	0.00	0.00	0.00	1.00	E African durras
SC70-14E	0.37	0.22	0.22	0.13	0.06	mixed
SC700-14E	0.48	0.23	0.15	0.07	0.07	mixed
SC701-14E	0.90	0.01	0.04	0.04	0.02	mixed
SC702-14E	0.71	0.01	0.08	0.15	0.05	mixed
SC704-14E	0.76	0.22	0.02	0.00	0.00	mixed
SC705-14E	0.78	0.05	0.10	0.03	0.03	mixed
SC707-14E	0.86	0.01	0.11	0.00	0.03	mixed
SC708-14E	0.94	0.02	0.02	0.01	0.00	mixed
SC709-14E	0.44	0.03	0.44	0.08	0.00	mixed
SC712-14E	0.85	0.07	0.00	0.06	0.02	mixed
SC715-14E	0.88	0.03	0.00	0.06	0.03	mixed
SC716-14E	0.00	0.50	0.50	0.00	0.00	mixed
SC719-14E	0.62	0.09	0.15	0.10	0.05	mixed
SC72-9	0.35	0.21	0.26	0.08	0.10	mixed
SC721-14E	0.23	0.04	0.68	0.05	0.00	mixed
SC723-14E	0.81	0.09	0.01	0.04	0.04	mixed
SC724-14E	0.98	0.01	0.00	0.00	0.01	Caudatum
SC725-14E	0.74	0.08	0.09	0.08	0.00	mixed
SC726-14E	0.94	0.03	0.01	0.01	0.01	mixed
SC727-14E	0.91	0.02	0.06	0.01	0.00	mixed
SC728-14E	0.84	0.06	0.00	0.09	0.01	mixed
SC730-14E	0.98	0.01	0.01	0.00	0.00	Caudatum
SC733-14E	0.82	0.08	0.03	0.04	0.04	mixed
SC734-14E	0.03	0.00	0.93	0.04	0.00	mixed
SC736-14E	0.32	0.02	0.63	0.01	0.02	mixed
SC737-14E	0.94	0.02	0.00	0.02	0.02	mixed
SC738-14E	0.70	0.04	0.14	0.10	0.03	mixed
SC74-3	0.06	0.01	0.74	0.16	0.02	mixed
SC741-14E	0.00	0.02	0.11	0.87	0.01	mixed
SC748-14E	0.83	0.00	0.13	0.04	0.00	mixed
SC749-14E	0.98	0.01	0.00	0.01	0.01	Caudatum
SC751-14E	0.00	0.37	0.63	0.00	0.00	mixed
SC752-14	0.11	0.02	0.09	0.72	0.06	mixed
SC753-3	0.36	0.00	0.57	0.07	0.00	mixed
SC754-14	0.94	0.00	0.03	0.03	0.00	mixed

SC755-14E	0.12	0.16	0.36	0.17	0.19	mixed
SC756-14E	0.04	0.00	0.03	0.90	0.02	mixed
SC757-14E	0.08	0.05	0.00	0.70	0.17	mixed
SC759-6	0.68	0.00	0.24	0.08	0.00	mixed
SC760-14E	0.86	0.03	0.06	0.05	0.00	mixed
SC761-14E	0.00	0.00	1.00	0.00	0.00	Kafir
SC762-14	0.97	0.02	0.00	0.00	0.02	Caudatum
SC763-14E	0.77	0.01	0.22	0.00	0.00	mixed
SC764-14E	0.80	0.01	0.20	0.00	0.00	mixed
SC77-14E	0.24	0.18	0.40	0.11	0.07	mixed
SC770-14E	0.89	0.04	0.00	0.04	0.03	mixed
SC773-14E	0.04	0.06	0.10	0.65	0.16	mixed
SC774-14	0.88	0.01	0.05	0.03	0.03	mixed
SC779-14E	0.17	0.04	0.74	0.05	0.00	mixed
SC78-14E	0.23	0.17	0.42	0.10	0.08	mixed
SC780-14E	0.87	0.01	0.05	0.05	0.02	mixed
SC781-14E	0.14	0.04	0.04	0.75	0.04	mixed
SC782-14E	0.05	0.11	0.19	0.00	0.66	mixed
SC784-14E	0.01	0.04	0.07	0.88	0.00	mixed
SC787-14E	0.83	0.02	0.09	0.06	0.00	mixed
SC79-14E	0.23	0.18	0.39	0.12	0.08	mixed
SC790-6	0.62	0.00	0.34	0.04	0.00	mixed
SC797-14E	0.04	0.02	0.94	0.00	0.00	mixed
SC798-14E	0.99	0.00	0.01	0.00	0.00	Caudatum
SC80-14E	0.61	0.04	0.14	0.16	0.06	mixed
SC800-14E	1.00	0.00	0.00	0.00	0.00	Caudatum
SC803-14E	0.96	0.01	0.00	0.03	0.00	Caudatum
SC804-14E	0.98	0.00	0.00	0.01	0.00	Caudatum
SC805-14E	0.95	0.00	0.03	0.01	0.01	mixed
SC807-14E	0.04	0.08	0.13	0.74	0.01	mixed
SC808-14E	0.58	0.05	0.25	0.10	0.01	mixed
SC810-14E	0.89	0.00	0.11	0.00	0.00	mixed
SC814-9	0.00	0.00	0.36	0.64	0.00	mixed
SC817-14E	0.33	0.07	0.24	0.30	0.05	mixed
SC819-14E	0.00	0.01	0.02	0.97	0.00	Asian durras
SC821-14E	0.00	0.00	1.00	0.00	0.00	Kafir
SC823-14E	0.00	0.00	0.00	0.00	1.00	E African durras
SC826-14E	0.06	0.01	0.00	0.93	0.00	mixed
SC827-14E	0.08	0.01	0.04	0.85	0.02	mixed
SC83-14E	0.26	0.29	0.31	0.05	0.10	mixed
SC830-14E	0.05	0.02	0.07	0.83	0.02	mixed
SC831-14E	0.00	0.00	0.03	0.97	0.00	Asian durras
SC832-14E	0.01	0.01	0.00	0.99	0.00	Asian durras
SC833-13E	0.00	0.00	0.03	0.97	0.00	Asian durras
SC834-13E	0.05	0.01	0.00	0.92	0.02	mixed
SC837-6	0.00	0.00	0.24	0.76	0.00	mixed
SC839-14E	0.00	0.00	0.06	0.93	0.00	mixed

SC84-14E	0.29	0.29	0.30	0.03	0.09	mixed
SC841-14E	0.00	0.00	0.48	0.48	0.04	mixed
SC842-14E	0.08	0.13	0.35	0.22	0.23	mixed
SC846-14	0.98	0.00	0.00	0.02	0.00	Caudatum
SC847-13E	0.00	0.01	0.04	0.95	0.00	mixed
SC848-13	0.00	0.00	0.01	0.99	0.00	Asian durras
SC85-6	0.42	0.19	0.25	0.07	0.07	mixed
SC851-14E	0.00	0.00	0.83	0.13	0.04	mixed
SC852-14E	0.12	0.08	0.08	0.59	0.14	mixed
SC854-6	0.00	0.00	0.42	0.58	0.00	mixed
SC855-14E	0.13	0.10	0.17	0.43	0.17	mixed
SC858-3	0.00	0.00	0.50	0.50	0.00	mixed
SC859-14E	0.02	0.02	0.01	0.96	0.00	Asian durras
SC86-14E	0.29	0.27	0.32	0.06	0.06	mixed
SC863-14E	0.00	0.00	1.00	0.00	0.00	Kafir
SC865-14E	0.01	0.00	0.16	0.82	0.01	mixed
SC868-3	0.23	0.00	0.63	0.13	0.01	mixed
SC87-14E	0.15	0.16	0.65	0.00	0.04	mixed
SC871-6	0.00	0.00	0.18	0.82	0.00	mixed
SC875-14E	0.00	0.00	1.00	0.00	0.00	Kafir
SC876-14E	0.00	0.00	0.00	1.00	0.00	Asian durras
SC877-3	0.00	0.00	0.48	0.52	0.00	mixed
SC888-14E	0.01	0.00	0.01	0.98	0.00	Asian durras
SC891-14E	0.03	0.00	0.00	0.97	0.00	Asian durras
SC893-3	0.00	0.00	0.56	0.44	0.00	mixed
SC895-3	0.00	0.00	0.29	0.71	0.00	mixed
SC90-14E	0.40	0.26	0.12	0.13	0.08	mixed
SC902-14E	0.03	0.01	0.95	0.00	0.00	Kafir
SC905-14E	0.03	0.00	0.04	0.92	0.00	mixed
SC906-14E	0.95	0.00	0.00	0.05	0.00	mixed
SC91-14E	0.03	0.93	0.01	0.00	0.03	mixed
SC910-14E	0.00	0.00	1.00	0.00	0.00	Kafir
SC913-14E	0.52	0.03	0.45	0.00	0.00	mixed
SC919-14E	0.04	0.01	0.02	0.93	0.00	mixed
SC921-3	0.00	0.00	0.41	0.59	0.00	mixed
SC923-3	0.00	0.00	0.45	0.55	0.00	mixed
SC924-14E	0.00	0.00	0.12	0.88	0.00	mixed
SC929-14E	0.00	0.00	0.00	1.00	0.00	Asian durras
SC93-14E	0.73	0.09	0.04	0.12	0.03	mixed
SC935-14E	0.22	0.08	0.08	0.53	0.09	mixed
SC94-14E	0.00	0.98	0.02	0.00	0.00	Guinea
SC941-14E	0.07	0.05	0.53	0.15	0.20	mixed
SC942-14E	0.00	0.00	0.00	0.00	0.99	E African durras
SC947-13E	0.57	0.25	0.05	0.09	0.05	mixed
SC949-14E	0.00	0.01	0.82	0.12	0.04	mixed
SC950-14E	0.02	0.01	0.88	0.09	0.00	mixed
SC951-14E	0.03	0.13	0.20	0.49	0.15	mixed

SC956-14E	0.00	0.86	0.11	0.01	0.02	mixed
SC96-14E	0.77	0.10	0.02	0.03	0.07	mixed
SC963-14E	0.85	0.06	0.06	0.00	0.03	mixed
SC964-14E	0.97	0.00	0.03	0.00	0.00	Caudatum
SC969-14E	0.37	0.23	0.28	0.06	0.05	mixed
SC97-14	0.00	1.00	0.00	0.00	0.00	#N/A
SC97-14E	0.00	1.00	0.00	0.00	0.00	Guinea
SC971-14E	0.40	0.15	0.35	0.02	0.07	mixed
SC972-14E	0.98	0.00	0.00	0.02	0.00	Caudatum
SC975-14E	0.13	0.12	0.25	0.26	0.25	mixed
SC979-14E	0.96	0.00	0.04	0.00	0.00	Caudatum
SC98-14E	0.00	1.00	0.00	0.00	0.00	Guinea
SC982-14	0.98	0.02	0.00	0.00	0.00	Caudatum
SC987-14E	0.03	0.05	0.18	0.13	0.61	mixed
SC99-14E	0.88	0.00	0.06	0.03	0.03	mixed
SC998-14E	0.12	0.10	0.16	0.49	0.12	mixed
SC999-14E	0.00	0.00	0.00	0.00	1.00	E African durras
IS13848	0.97	0.01	0.00	0.00	0.02	Caudatum
IS22287	0.04	0.00	0.60	0.27	0.09	mixed
IS25733	0.11	0.84	0.00	0.04	0.01	mixed
IS27390	0.10	0.66	0.00	0.13	0.11	mixed
IS8525	0.00	0.00	1.00	0.00	0.00	Kafir
Karper 669	0.07	0.46	0.33	0.12	0.03	mixed
Kuyuma	0.99	0.00	0.01	0.00	0.00	Caudatum
L1999B-5	0.87	0.00	0.13	0.00	0.00	mixed
LR9198	0.05	0.00	0.11	0.80	0.04	mixed
Macia	0.99	0.00	0.01	0.00	0.00	Caudatum
PI525695	0.01	0.49	0.14	0.03	0.34	mixed
PI563516	0.07	0.39	0.52	0.03	0.00	mixed
PI609477	0.09	0.89	0.00	0.00	0.02	mixed
PI656046	0.12	0.02	0.23	0.57	0.06	mixed
QL12	0.00	0.51	0.43	0.04	0.02	mixed
R9188	0.38	0.00	0.62	0.00	0.00	mixed
R9247	0.44	0.00	0.51	0.04	0.00	mixed
R931945-2-2	0.66	0.00	0.26	0.07	0.01	mixed
R986087-2-4-1	0.69	0.00	0.23	0.04	0.03	mixed
R993396	0.65	0.00	0.18	0.14	0.02	mixed
R995248	0.62	0.03	0.14	0.17	0.04	mixed
SC1012-8BK	1.00	0.00	0.00	0.00	0.00	Caudatum
SC1013-11Ebk	0.99	0.00	0.00	0.01	0.00	Caudatum
SC1018-11Ebk	0.85	0.04	0.00	0.08	0.03	mixed
SC1047-11Ebk	0.06	0.07	0.05	0.66	0.16	mixed
SC1048-8bk	0.10	0.01	0.16	0.59	0.14	mixed
SC1053-8bk	0.08	0.05	0.10	0.63	0.15	mixed
SC1061-8bk	0.20	0.01	0.76	0.03	0.00	mixed
SC1068-11Ebk	0.73	0.01	0.21	0.04	0.01	mixed
SC1075-8bk	0.05	0.79	0.11	0.05	0.00	mixed

SC1079-11Ebk	0.89	0.03	0.01	0.05	0.02	mixed
SC1081-8bk	0.00	0.88	0.12	0.00	0.00	mixed
SC1106-8bk	0.01	0.00	0.10	0.00	0.89	mixed
SC1108-11Ebk	0.11	0.15	0.57	0.15	0.03	mixed
SC1112-8bk	0.81	0.19	0.00	0.00	0.00	mixed
SC1113-11Ebk	0.69	0.09	0.07	0.09	0.06	mixed
SC1115-11Ebk	0.20	0.59	0.08	0.13	0.00	mixed
SC1119-8bk	0.00	0.87	0.10	0.01	0.01	mixed
SC1120-8bk	0.37	0.18	0.25	0.16	0.04	mixed
SC1136-11Ebk	0.52	0.11	0.27	0.05	0.05	mixed
SC1139-5bk	0.77	0.00	0.22	0.01	0.00	mixed
SC1152-5bk	0.25	0.07	0.31	0.29	0.08	mixed
SC1156-11Ebk	0.03	0.10	0.02	0.66	0.19	mixed
SC1157-11Ebk	0.05	0.10	0.05	0.63	0.17	mixed
SC1159-11Ebk	0.11	0.08	0.02	0.64	0.16	mixed
SC1162-8bk	0.07	0.00	0.13	0.00	0.80	mixed
SC1174-5bk	0.00	0.00	0.21	0.00	0.79	mixed
SC1175-5bk	0.00	0.00	0.37	0.23	0.40	mixed
SC1185-11Ebk	0.23	0.15	0.37	0.21	0.04	mixed
SC1189-8bk	0.00	0.00	0.10	0.12	0.77	mixed
SC1190-5bk	0.02	0.00	0.40	0.45	0.12	mixed
SC1192-8bk	0.38	0.00	0.28	0.27	0.07	mixed
SC1196-8bk	0.05	0.03	0.22	0.65	0.04	mixed
SC1197-8bk	0.01	0.03	0.32	0.56	0.08	mixed
SC1205-5bk	0.84	0.11	0.00	0.03	0.01	mixed
SC1209-5bk	0.18	0.15	0.66	0.00	0.01	mixed
SC1212-5bk	0.81	0.00	0.19	0.00	0.00	mixed
SC1220-11Ebk	0.53	0.16	0.12	0.14	0.05	mixed
SC1222-11Ebk	0.57	0.13	0.12	0.13	0.06	mixed
SC1226-2bk	0.33	0.08	0.48	0.10	0.02	mixed
SC1227-2bk	0.00	0.50	0.45	0.05	0.00	mixed
SC1228-2bk	0.38	0.00	0.55	0.06	0.00	mixed
SC1230-5bk	0.46	0.10	0.24	0.17	0.03	mixed
SC1233-2bk	0.00	0.65	0.35	0.00	0.00	mixed
SC1234-2bk	0.36	0.01	0.49	0.11	0.03	mixed
SC1236-2bk	0.01	0.00	0.91	0.08	0.00	mixed
SC1241-2bk	0.40	0.22	0.27	0.07	0.04	mixed
SC1244-11Ebk	0.47	0.17	0.25	0.09	0.03	mixed
SC1246-11Ebk	0.69	0.10	0.05	0.14	0.03	mixed
SC1251-2bk	0.05	0.00	0.92	0.04	0.00	mixed
SC1252-5bk	0.05	0.01	0.27	0.62	0.06	mixed
SC1255-8bk	0.91	0.01	0.07	0.01	0.00	mixed
SC1256-8bk	0.44	0.09	0.17	0.23	0.06	mixed
SC1258-8bk	0.73	0.00	0.26	0.01	0.00	mixed
SC1260-5bk	0.46	0.00	0.51	0.03	0.00	mixed
SC1264-5bk	0.67	0.20	0.09	0.04	0.00	mixed
SC1267-8bk	0.73	0.17	0.08	0.02	0.00	mixed

SC1268-5bk	0.74	0.00	0.26	0.00	0.00	mixed
SC1274-8bk	1.00	0.00	0.00	0.00	0.00	Caudatum
SC1278-5bk	0.21	0.00	0.24	0.55	0.00	mixed
SC1280-5bk	0.62	0.01	0.24	0.11	0.02	mixed
SC1281-5bk	0.77	0.00	0.20	0.03	0.00	mixed
SC1282-5bk	0.54	0.02	0.20	0.18	0.05	mixed
SC1286-2bk	0.44	0.00	0.47	0.09	0.00	mixed
SC1288-5bk	0.27	0.00	0.73	0.00	0.00	mixed
SC1290-5bk	0.85	0.00	0.15	0.00	0.00	mixed
SC1291-5bk	0.70	0.00	0.30	0.00	0.00	mixed
SC1294-8bk	0.36	0.01	0.63	0.00	0.00	mixed
SC1295-8bk	0.45	0.31	0.15	0.10	0.00	mixed
SC1296-5bk	0.09	0.19	0.67	0.01	0.05	mixed
SC1297-5bk	0.31	0.00	0.69	0.01	0.00	mixed
SC1301-5bk	0.65	0.00	0.35	0.00	0.00	mixed
SC1303-2bk	0.54	0.00	0.40	0.06	0.00	mixed
SC1304-5bk	0.74	0.00	0.24	0.02	0.00	mixed
SC1308-2bk	0.00	0.00	0.53	0.41	0.06	mixed
SC1309-5bk	0.65	0.00	0.35	0.00	0.00	mixed
SC1323-5bk	0.16	0.02	0.33	0.39	0.10	mixed
SC1326-5bk	0.19	0.11	0.27	0.32	0.11	mixed
SC1327-2bk	0.06	0.02	0.59	0.29	0.04	mixed
SC133-8BK	0.09	0.02	0.09	0.50	0.30	mixed
SC1338-11Ebk	0.07	0.72	0.14	0.05	0.02	mixed
SC1340-2bk	0.50	0.23	0.25	0.00	0.02	mixed
SC1343-2bk	0.01	0.38	0.54	0.07	0.00	mixed
SC1344-2bk	0.56	0.00	0.44	0.00	0.00	mixed
SC1347-2bk	0.15	0.07	0.38	0.34	0.05	mixed
SC1348-5bk	0.52	0.06	0.27	0.10	0.05	mixed
SC1349-5bk	0.47	0.09	0.23	0.16	0.04	mixed
SC1350-5bk	0.42	0.14	0.26	0.14	0.04	mixed
SC1352-5bk	0.42	0.04	0.39	0.11	0.04	mixed
SC1353-5bk	0.49	0.13	0.19	0.16	0.03	mixed
SC1355-5bk	0.46	0.13	0.19	0.18	0.03	mixed
SC1359-2bk	0.49	0.00	0.47	0.03	0.00	mixed
SC1360-2bk	0.11	0.12	0.43	0.29	0.05	mixed
SC1361-5bk	0.37	0.13	0.30	0.17	0.03	mixed
SC1362-2bk	0.65	0.10	0.25	0.00	0.00	mixed
SC1368-2bk	0.62	0.10	0.21	0.06	0.00	mixed
SC1369-2bk	0.73	0.13	0.10	0.04	0.00	mixed
SC1371-2bk	0.76	0.00	0.24	0.00	0.00	mixed
SC1374-2bk	0.42	0.12	0.46	0.00	0.00	mixed
SC1375-2bk	0.76	0.03	0.20	0.01	0.00	mixed
SC1377-2bk	0.31	0.18	0.46	0.03	0.02	mixed
SC1380-11Ebk	0.51	0.10	0.23	0.14	0.02	mixed
SC1381-2bk	0.52	0.06	0.42	0.00	0.00	mixed
SC1382-2bk	0.24	0.00	0.73	0.03	0.00	mixed

SC1386-5bk	0.26	0.01	0.67	0.06	0.00	mixed
SC1387-2bk	0.38	0.01	0.57	0.04	0.00	mixed
SC1388-2bk	0.43	0.00	0.57	0.00	0.00	mixed
SC1390-2bk	0.73	0.00	0.27	0.00	0.00	mixed
SC1392-5bk	0.10	0.06	0.04	0.64	0.15	mixed
SC1393-2bk	0.54	0.10	0.31	0.04	0.01	mixed
SC1394-2bk	0.78	0.00	0.22	0.00	0.00	mixed
SC1395-2bk	0.50	0.07	0.41	0.00	0.01	mixed
SC1396-2bk	0.50	0.06	0.41	0.02	0.00	mixed
SC1397-2bk	0.46	0.09	0.40	0.05	0.01	mixed
SC1398-2bk	0.42	0.07	0.48	0.04	0.00	mixed
SC1410-2bk	0.10	0.04	0.51	0.32	0.04	mixed
SC1411-2bk	0.09	0.06	0.47	0.30	0.07	mixed
SC1412-2bk	0.10	0.04	0.55	0.26	0.05	mixed
SC1413-2bk	0.11	0.05	0.55	0.25	0.04	mixed
SC1415-2bk	0.10	0.02	0.51	0.30	0.07	mixed
SC1421-2bk	0.05	0.05	0.38	0.46	0.06	mixed
SC1423-2bk	0.00	0.05	0.53	0.36	0.06	mixed
SC1430-2bk	0.32	0.00	0.59	0.08	0.02	mixed
SC1431-2bk	0.41	0.00	0.52	0.08	0.00	mixed
SC219-8BK	0.00	0.00	0.19	0.81	0.00	mixed
SC355-8BK	0.27	0.52	0.13	0.08	0.00	mixed
SC400-5BK	0.00	0.28	0.30	0.42	0.00	mixed
C45-14TempB	0.00	0.02	0.02	0.25	0.71	mixed
SC513-11EBK	0.08	0.22	0.56	0.13	0.01	mixed
SC610-5BK	0.01	0.00	0.35	0.57	0.08	mixed
SC674-5BK	0.73	0.00	0.26	0.02	0.00	mixed
SC676-5BK	0.34	0.00	0.66	0.00	0.00	mixed
SC678-5BK	0.80	0.00	0.20	0.00	0.00	mixed
SC688-8BK	0.88	0.00	0.12	0.00	0.00	mixed
SC711-11EBK	0.73	0.04	0.21	0.03	0.00	mixed
SC758-8BK	0.72	0.04	0.22	0.02	0.00	mixed
SC809-11EBK	0.72	0.00	0.26	0.03	0.00	mixed
SC824-8BK	0.00	0.05	0.53	0.34	0.09	mixed
SC843-8BK	0.00	0.00	0.42	0.58	0.00	mixed
SC844-8BK	0.15	0.00	0.08	0.76	0.01	mixed
SC850-8BK	0.25	0.00	0.13	0.62	0.00	mixed
SC916-11EBK	0.23	0.04	0.18	0.43	0.11	mixed
SC92-4	0.00	0.00	0.61	0.36	0.03	mixed
SC961-2BK	0.00	0.00	0.21	0.79	0.00	mixed
SC962-2BK	0.00	0.00	0.08	0.92	0.00	mixed
Sureno	1.00	0.00	0.00	0.00	0.00	Caudatum
