# Supplementary material for: Large‐scale GWAS in sorghum reveals common genetic control of grain size among cereals
Source: Plant Biotechnol J. 2019 Nov 11;18(4):1093–105. doi: 10.1111/pbi.13284 (PMC7061873; doi:10.1111/pbi.13284)
Supplement: Supplementary file 7 — Table S6 List of reported grain size genes in rice and maize. [file PBI-18-1093-s002.pdf]

Table S6 Lis of reported grain size genes in rice and maize

Species	Gene name	Encoded protein	Methods	Gene ID	Reference
Maize	Bt2	AGP small subunit	Transgenic experiment	Zm00001d050032	Jiang et al. 2013
Maize	CYP724B3	CYP724B3	Transgenic experiment	Zm00001d003349	Wu et al. 2008
Maize	GbssIIa	UDP-Glycosyltransferase superfamily protein	Transgenic experiment	Zm00001d019479	Jiang et al. 2013
Maize	Gln-4	Glutamine synthetase isoenzymes	Mutant analysis	Zm00001d051804	Martin et al. 2006
Maize	Mn1	NA	Mutant analysis	Zm00001d003776	Miller 1992
Maize	O1	myosin XI motor protein	Mutant analysis	Zm00001d052110	Wang et al. 2012
Maize	O2	Regulatory protein opaque-2	Mutant analysis	Zm00001d018971	Hartings et al. 1989
Maize	PBF1	endosperm-specific transcription factor	NIL analysis	Zm00001d005100	Lang et al. 2014
Maize	SbeI	Uncharacterized protein	Transgenic experiment	Zm00001d036361	Jiang et al. 2013
Maize	SbeIIb	1,4-alpha-glucan-branching enzyme	Transgenic experiment	Zm00001d016684	Jiang et al. 2013
Maize	Sh1	Sucrose synthase 1	Transgenic experiment	Zm00001d045042	Jiang et al. 2013
Maize	Sh2	AGP large subunit	tant analysis/Transgenic experir	Zm00001d044129	Jiang et al. 2013
Maize	SMK1	pentatricopeptide repeat protein	Mutant analysis	Zm00001d007100	Li et al. 2014
Maize	ZmIPT2	cytokinin biosynthetic enzyme	Association study	Zm00001d003869	Weng et al. 2013
Maize	ZmSWEET4c	Transepithelial hexose transporter	Mutant analysis	Zm00001d015912	Sosso et al. 2015
Rice	DEP2	novel plant-specific protein	QTL Cloning	Os07g0616000	Li et al. 2010
Rice	An-1	a basic helix-loop-helix protein	QTL Cloning	Os04g0350700	Luo et al. 2013
Rice	APG	Typical bHLH protein	Transgenic experiment	Os05g0139100	Heang and Sassa 2012
Rice	BC14	Golgi-localized nucleotide sugar transporters	Mutant analysis	Os02g0614100	Zhang et al. 2011
Rice	BG1	Primary auxin response gene	Mutant analysis	Os03g0175800	Liu et al. 2015
Rice	BG2	Cytochrome P450	Transgenic experiment	Os07g0603700	Xu et al. 2015
Rice	*BG3*	a plasma membrane transporter of cytokinin	Mutant analysis	Os01g0680200	Xiao et al. 2018
Rice	BRD1	Brassinosteroid-6-oxidase	Mutant analysis	Os03g0602300	Mori et al. 2002
Rice	BRD2	homologous to Arabidopsis DIM1/DWF1	Mutant analysis	Os10g0397400	Hong et al. 2005
Rice	Bu1	a helix-loop-helix protein	Transgenic experiment	Os06g0226500	Tanaka et al. 2009
Rice	Cyc-T1;3	Cyclin-T1;3	Transgenic experiment	Os11g0157100	Qi et al. 2012
Rice	CYP704A3	a putative cytochrome P450	Transgenic experiment	Os04g0573900	Tang et al. 2016
Rice	CYP90B2	CYP90B2	Transgenic experiment	Os03g0227700	Wu et al. 2008
Rice	D11	Novel Cytochrome P450	Mutant analysis	Os04g0469800	Tanabe et al. 2005
Rice	D2	CYP90D2	Mutant analysis	Os01g0197100	Hong et al. 2003
Rice	D61	OsBRI1	Mutant analysis	Os01g0718300	Morinaka et al. 2006
Rice	DEP1	PEBP-like domain protein	QTL Cloning	Os09g0441900	Huang et al. 2009
Rice	DEP3	Patatin-like phospholipase A2	Association study	Os06g0677000	Qiao et al. 2011
Rice	DLT	GRAS family protein	Transgenic experiment	Os06g0127800	Tong et al. 2012
Rice	FLO2	Protein with a tetratricopeptide repeat motif	Mutant analysis	Os04g0645100	She et al. 2010
Rice	FUWA	NHL domain-containing protein	Transgenic experiment	Os02g0234200	Chen et al. 2015
Rice	GAD1	DERMAL PATTERNING FAC- TOR-LIKE pe	QTL Cloning	Os08g0485500	Jin et al. 2016
Rice	*GGC2*	Gγ protein	Transgenic experiment	Os08g0456600	Sun et al. 2018
Rice	GIF1	Cell-wall invertase	Mutant analysis	Os04g0413500	Wang et al. 2008
Rice	GL3.1/qGL3	Phosphatase with Kelch-like repeat domain	QTL Cloning	Os03g0646900	Qi et al. 2012; Zhang 2012
Rice	GL4	Myb-like protein	QTL Cloning	ORGLA04G025430C	Wu et al. 2017
Rice	GLW7	Plant-specific transcription factor OsSPL13	QTL Cloning	Os07g0505200	Si et al. 2016
Rice	Grain Length3.2	Cytochrome P450	Transgenic experiment	Os03g0417700	Xu et al. 2015
Rice	GS2/GL2	Growth regulating factor 4	QTL Cloning	Os02g0701300	Che et al. 2015; Hu et al. 2015

Rice	GS3	Trans-membrane protein	QTL Cloning	OS03G0407400	Mao et al. 2010
Rice	GS5	Putative serine carboxypeptidase	QTL Cloning	Os05g0158500	Li et al. 2011
Rice	GS9	an unknown expressed protein	QTL Cloning	LOC_Os09g27590	Zhao et al. 2018
Rice	GSK2	GSK3/SHAGGY-like; regulator of BR signaling kinase	Transgenic experiment	Os05g0207500	Tong et al. 2012
Rice	*gsn1/LARGE8*	OsMKP1	Mutant analysis	Os05g0115800	Guo et al. 2018; Xu etal., 2018
Rice	GW2	RING-type E3 ubiquitin ligase	QTL Cloning	Os02g0244100	Song et al. 2007
Rice	GW5/qSW5	calmodulin binding protein	QTL Cloning	Os05g0187500	Liu et al. 2017
Rice	GW6	GNAT-like protein	QTL	Os06g0650300	Song et al. 2015
Rice	GW8/OsPL16	Squamosa promoter-binding protein like 16	QTL Cloning	Os08g0531600	Wang et al. 2012
Rice	HGW	ubiquitin-associated domain protein	Mutant analysis	Os06g0160400	Li et al. 2012
Rice	MHZ7	a membrane protein homologous to EIN2	Mutant analysis	Os07g0155600	Ma et al. 2013
Rice	IYB transcription fa	A MYB transcription factor	Association study	Os07G0497500	Huang et al. 2012
Rice	A Zinc finger protei	A Zinc finger protein	Association study	Os02G0192300	Huang et al. 2012
Rice	A Expressed protei	A Expressed protein	Association study	Os03G0604566	Huang et al. 2012
Rice	A transport protein	A transport protein	Association study	Os03G0604600	Huang et al. 2012
Rice	A Expressed protei	A Expressed protein	Association study	Os03G0574600	Huang et al. 2012
Rice	Nuf2 family protei	A Nuf2 family protein	Association study	Os03G0577100	Huang et al. 2012
Rice	Nuf2 family protei	A Nuf2 family protein	Association study	Os03G0577100	Huang et al. 2012
Rice	Nuf2 family protei	A Nuf2 family protein	Association study	Os03G0577100	Huang et al. 2012
Rice	A GASR7	A GASR7	Association study	Os06G0266800	Huang et al. 2012
Rice	A transcription facto	A transcription factor	Association study	Os06G0265400	Huang et al. 2012
Rice	Areceptor-like kinas	Areceptor-like kinase	Association study	Os07G0501800	Huang et al. 2012
Rice	OsAGSW1	Chloroplast-localized ABC1 protein kinase	Transgenic experiment	Os05g0323800	Li et al. 2015
Rice	*OsARF4*	an auxin response factor (ARF) family protein	Transgenic experiment	Os01g0927600	Hu et al. 2018
Rice	OsBAK1	Leucine-rich repeat receptor-like kinase	Mutant analysis	Os08g0174700	Yuan et al. 2017
Rice	OsBUL1	bHLH transcriptional activator	Transgenic experiment	Os02g0747900	Jang and Li 2017
Rice	OsBZR1	Transcription factor mediating BRresponses	Transgenic experiment	Os07g0580500	Zhu et al. 2015
Rice	OsCCS52B	rice cell cycle switch 52 B	Mutant analysis	Os01g0972900	Su'udi et al. 2012
Rice	OsFBK12	F-box protein containing a Kelch repeat motif	Transgenic experiment	Os03g0171600	Chen et al. 2013
Rice	OsFIE1	Putative Polycomb group protein FIE2	Transgenic experiment	Os08g0137250	Folsom et al. 2014
Rice	OsFIE2	Putative Polycomb group protein FIE2	Transgenic experiment	Os08g0137100	Na et al. 2012
Rice	OsGIF1	Rice GRF-interacting protein 1	Transgenic experiment	Os03g0733600	He et al. 2017
Rice	OsMAPK6	Mitogen-activated protein kinase 6	Mutant analysis	Os06g0154500	Liu et al. 2015b
Rice	*OsMKK4*	MKK4	Mutant analysis	Os02g0787300	Xu etal., 2018
Rice	*OsMKKK10*	MKKK10	Mutant analysis	Os04g0559800	Xu etal., 2018
Rice	*OsMPK6*	MPK6	Mutant analysis	Os10g0533600	Guo et al. 2018
Rice	OsPPKL2	Phosphatase with Kelch-like repeat domain	Transgenic experiment	Os05g0144400	Zhan et al. 2012
Rice	OsPPKL3	Phosphatase with Kelch-like repeat domain	Transgenic experiment	Os12g0617900	Zhan et al. 2012
Rice	*ospup7*	a plasma membrane transporter of cytokinin	Transgenic experiment	Os05g0556800	Xiao et al. 2018
Rice	OsSAMS1	-ADENOSYL-L-METHIONINE SYNTHETASI	Transgenic experiment	Os05g0135700	Chen et al. 2013
Rice	OsSGL	DUF1645 family protein	Transgenic experiment	Os02g0134200	Wang et al. 2016
Rice	*OsSPMS1*	a SPMS encoding gene	Transgenic experiment	Os06g0528600	Tao etal. 2018
Rice	OsSUT2	Sucrose transporter	Mutant analysis	Os12g0641400	Eom et al. 2011
Rice	PGL1	Atypical bHLH protein	Transgenic experiment	Os03g0171300	Heang and Sassa 2012
Rice	PGL2	Atypical bHLH protein	Transgenic experiment	Os02g0747900	Heang and Sassa 2012b
Rice	qGL3-2/qLGY3	MADS-box transcription factor 1	QTL Cloning	Os03g0215400	Liu etal. 2018; Yu etal., 2018

Rice	qGW7/GL7	TONNEAU1-recruiting motif protein	QTL Cloning	LOC_Os07g41200	Wang et al. 2015a; Wang et al. 2015b
Rice	*TGW3/GL3.3/TGW*	GSK3/SHAGGY-Like Kinase OsGSK5/OsSK41	QTL Cloning	Os03g0841800	u et a. 2018; Xiao et al. 2018; Ying et al. 201
Rice	RGA1/D1	heterotrimeric G protein α subunit	Mutant analysis	Os05g0333200	Ashikari 1999
Rice	RGB1	G protein β subunit	Transgenic experiment	Os03g0669100	Utsunomiya et al. 2011
Rice	RSR1	RICE STARCH REGULATOR1	Mutant analysis	Os05g0121600	Fu and Xue 2010
Rice	SDG725	H3K36 methyltransferase	Transgenic experiment	Os02g0554000	Sui et al. 2012
Rice	SERF1	transcription factor SALT-RESPONSIVE ERF1	Mutant analysis	Os05g0420300	Schmidt et al. 2013
Rice	SG1	Novel protein	Mutant analysis	Os09g0459200	Nakagawa et al. 2012
Rice	SGL1	Novel protein	Transgenic experiment	OS02G0762600	Nakagawa et al. 2012
Rice	SLG	BAHD acyltransferase-like protein	Mutant analysis	Os08g0562500	Feng et al. 2016
Rice	SMG1	mitogen-activated protein kinase kinase 4	Mutant analysis	Os02g0787300	Duan et al. 2014
Rice	SMOS1	ıusual APETALA2 (AP2)-type tran- scription fac	Mutant analysis	Os05g0389000	Hirano et al. 2017
Rice	SRS3	Kinesin 13 protein	Mutant analysis	Os05g0154700	Kitagawa et al. 2010
Rice	SRS5	Alpha-tubulin protein	Mutant analysis	Os11g0247300	Segami 2012
Rice	TGW6	Iindole-3-acetic acid (IAA)-glucose hydrolase	QTL Cloning	Os06g0623700	Ishimaru et al. 2013
Rice	TH1	a DUF640 domain-like gene	Mutant analysis	Os02g0811000	Li et al. 2012c
Rice	TIFY 11b	TIFY gene	Transgenic experiment	Os03g0181100	Hakata et al. 2012
Rice	TUD1	U-box E3 ubiquitin ligase	Mutant analysis	Os03g0232600	Hu et al. 2013
Rice	WTG1	Deubiquitinating enzyme	Mutant analysis	Os08g0537800	Wang et al. 2017
