# Supplementary material for: Large‐scale GWAS in sorghum reveals common genetic control of grain size among cereals
Source: Plant Biotechnol J. 2019 Nov 11;18(4):1093–105. doi: 10.1111/pbi.13284 (PMC7061873; doi:10.1111/pbi.13284)
Supplement: Supplementary file 8 — Table S7 Effect of population structure on grain size. Statistical significance was assessed using one‐way analyses of variance (ANOVAs) followed by Tukey’s HSD tests for multiple comparisons [file PBI-18-1093-s003.pdf]

Table S7 Effect of population structure on grain size

Racial groups	TKW	Volume	Length	Width	Thickness
E African durras	20.03[a]	21.10[a]	4.06[a]	3.11[a]	3.19[a]
Asian durras	25.57[b]	24.11[b]	4.24[b]	3.37[b]	3.20[a]
Kafir	27.47[b]	25.59[b]	4.42[c]	3.41[b]	3.23[a]
Guinea	31.22[c]	27.98[c]	4.71[d]	3.58[c]	3.16[a]
Caudatum	32.16[c]	27.84[c]	4.50[c]	3.62[c]	3.24[ab]
